# Dietary Behavior, Physical Activity, and 24-Hour Rhythm Coherence: A Digital Phenotyping Perspective on Vascular and Glycemic Health

**DOI:** 10.3390/nu18152546

**Published:** 2026-08-04

**Authors:** Guilong Sun, Xiangyu Jia, Hao Zhang, Ruida Yu, Siyu Rong, Yuyang Liu, Yufei Qi, Shengyi Chen

**Affiliations:** 1 School of Physical Education, Central-South Minzu University, Wuhan 430074, China; 2016022@mail.scuec.edu.cn; 2 School of Ethnology and Sociology, Central-South Minzu University, Wuhan 430074, China; 2025110098@mail.scuec.edu.cn; 3 Department of Physical Education and Research, Central South University, Changsha 410083, China; 230912026@csu.edu.cn (H.Z.); 240912003@csu.edu.cn (R.Y.); yufeiqi@csu.edu.cn (Y.Q.); 4 School of Physical Education, Hainan Normal University, Haikou 571158, China; yyangliu636@gmail.com; 5 Department of Health and Physical Education, The Education University of Hong Kong, Tai Po, Hong Kong, China; 6 School of Sports Economics and Management, Central University of Finance and Economics, Beijing 102206, China

**Keywords:** circadian rhythm, chrononutrition, physical activity timing, arterial stiffness, glycemic variability, wearable biosensors, time-restricted eating, cardiometabolic health

## Abstract

Background: Cardiometabolic risk is shaped not only by the amount of food intake and physical activity but also by their timing and regularity across the 24 h cycle. We propose rhythm coherence as a hypothesis-generating framework describing the temporal stability and alignment of eating, physical activity, and sleep. Its vascular and glycemic relevance has not yet been prospectively validated. Methods: This semi-structured narrative review was supported by a transparent, staged evidence-identification process using PubMed, Scopus, and Web of Science Core Collection. Main searches used a 2020–2026 window, with a broader 2018–2026 window for targeted athlete-focused searches. After cross-database deduplication, 1081 unique records entered broad screening; 694 underwent strict screening, yielding 257 core candidate records. A targeted athlete-focused search contributed 46 additional records, producing a 303-record candidate evidence pool for topic mapping. From this pool, 131 high-priority records underwent full-text narrative synthesis, and 92 sources were ultimately cited directly in the manuscript. These counts represent successive review stages rather than a PRISMA-defined systematic-review inclusion set. Results: Earlier or more regular eating patterns, postprandial physical activity, and stable sleep–wake schedules were associated with more favorable cardiometabolic profiles in selected studies. However, the evidence was heterogeneous and included mechanistic studies, observational analyses, and intervention trials. These findings support further investigation but do not establish an effect of integrated eating–activity–sleep coherence. Numerical effects reported for individual interventions should not be attributed to the proposed integrated framework or to the Rhythm Coherence Index (RCI). Conclusions: Coordinated assessment of eating, activity, and sleep timing may offer a useful research direction. The RCI is an author-proposed candidate composite, not an existing validated instrument or clinical decision rule. Its formula, component weights, missing-data procedures, thresholds, reliability, responsiveness, predictive value, and external validity require prospective evaluation before clinical application can be considered.

## 1. Introduction

### 1.1. Global Burden and Dual Determinants

Cardiovascular diseases and type 2 diabetes remain leading global causes of morbidity and mortality. Cardiovascular diseases alone account for more than 17 million deaths annually [1]. Traditional risk factors, including hypertension, dyslipidemia, and obesity, explain a substantial proportion of this burden. However, they do not fully account for inter-individual variation in cardiometabolic outcomes. Diet and physical activity are well-established modifiable risk factors [2,3]. However, their effects are heterogeneous. Individuals with similar macronutrient distributions and total daily activity levels may show markedly different vascular and glycemic outcomes. One critical dimension remains comparatively overlooked: the temporal organization of eating, physical activity, and rest within the 24 h cycle. Emerging evidence suggests that the timing and regularity of these behaviors may influence cardiometabolic health independently of their average levels. In this review, we propose rhythm coherence as a hypothesis-generating framework for examining whether the temporal stability and alignment of eating, physical activity, and sleep may be relevant to vascular and glycemic health. It is not presented as an established determinant or validated clinical construct.

### 1.2. The Average-Value Dilemma

The “average-value dilemma” captures a fundamental limitation of traditional nutrition and exercise epidemiology. Individuals with similar total daily energy intake or comparable daily moderate-to-vigorous physical activity (MVPA) may experience divergent cardiometabolic outcomes. Landmark studies have shown that inter-individual glycemic responses to standardized meals may vary five-fold or more [4]. Traditional metabolic markers explain only part of this variability. Meal timing and regularity explain additional variance in glycemic responses beyond total energy intake. This finding suggests that the temporal structure of eating may constitute a distinct risk dimension [5]. Similarly, compositional analyses of 24 h activity patterns may predict arterial stiffness more effectively than total MVPA alone [6]. These analyses account for the relative distribution of sedentary time, light physical activity, and MVPA across the day. These observations support a conceptual shift from how much a person eats or exercises to when, how regularly, and in what temporal relation these behaviors occur. The author-proposed rhythm coherence framework organizes this perspective for future testing. It hypothesizes that the alignment of eating, physical activity, and sleep rhythms may provide information beyond their average levels; this incremental and causal value remains to be established.

### 1.3. Digital Health Creates New Observational Windows

The convergence of wearable technologies and continuous monitoring devices has transformed the assessment of behavioral and physiological rhythms under free-living conditions. Continuous glucose monitoring (CGM) captures real-time glucose dynamics at 1–5 min resolution. Wrist actigraphy characterizes activity and sleep patterns over periods of seven days or longer. Digital meal logging through image recognition or ecological momentary assessment records meal timing and frequency. Ambulatory blood pressure monitors and cuffless wearables track 24 h cardiovascular dynamics. Emerging flexible biosensors can also measure sweat electrolytes, metabolic substrates, and stress-related hormones. Collectively, these tools enable multimodal digital phenotyping, defined here as the simultaneous measurement of eating, physical activity, sleep, and physiological rhythms within the same individual over weeks to months [7]. This transition from snapshot assessments, such as fasting glucose and office blood pressure, to continuous behavioral and physiological data streams has revealed previously hidden temporal patterns. These patterns include meal jitter, defined as day-to-day variability in eating times, as well as activity fragmentation and sleep–wake irregularity. All are increasingly quantifiable and potentially modifiable. The rhythm coherence framework leverages these observational windows to examine whether integrated analyses of multimodal behavioral rhythms can improve the prediction of cardiometabolic outcomes beyond isolated metrics.

### 1.4. Aim and Scope of This Review

This review has four objectives: (1) to synthesize evidence concerning the timing and regularity of eating, physical activity, and sleep in relation to vascular and glycemic outcomes; (2) to distinguish mechanistic plausibility, observational associations, and intervention evidence; (3) to describe digital phenotyping methods that could permit concurrent measurement of these behaviors; and (4) to propose rhythm coherence and a candidate Rhythm Coherence Index (RCI) as hypotheses for future validation. The review focuses on adults of both sexes across the physical activity spectrum, from sedentary individuals to recreationally active adults and highly trained athletes. Vascular endpoints include pulse wave velocity, flow-mediated dilation, and ambulatory blood pressure; glycemic endpoints include continuous-glucose-monitoring-derived time in range, coefficient of variation, and mean amplitude of glycemic excursions. The primary time scale is 24 h behavioral organization, with acute and longitudinal extensions considered in Section 7. The RCI is not treated as an established instrument, and no validated formula, weighting scheme, threshold, or clinical interpretation is currently available.

### 1.5. Review Methodology

Review design. This article is a semi-structured narrative review supported by a structured and transparent evidence-identification process. It was not preregistered and was not conducted as a systematic review or meta-analysis. Screening and data charting were not performed independently in duplicate, and formal risk-of-bias or certainty-of-evidence tools were not applied. Accordingly, the numerical flow reported below describes an evidence-identification process and candidate evidence pool rather than a PRISMA-defined set of included studies.

Information sources and search strategy. PubMed, Scopus, and Web of Science Core Collection were searched using concept strings combining eating, activity, sleep-rhythm, and digital-phenotyping terms with vascular and glycemic outcomes. Main searches used a 2020–2026 date window, and targeted athlete-focused searches used a 2018–2026 window. The retained source-tagged export files contained 771 PubMed records and 737 Web of Science records. Scopus records were searched and merged into the master citation library during the original workflow. To make the Scopus search transparent and visible, the strategy was rerun on 20 July 2026: the S1–S5 main-search strings yielded 4182 results in total across overlapping searches, of which 1122 records were exported, while the athlete-focused S6a–S6c searches yielded 368 results, of which 361 were exported. Because search strings overlap, these run-level totals are not treated as unique records or added arithmetically to the historical cohort. The complete database-specific strings and rerun ledger are reported in Supplementary File S1. Because the original source-level Scopus attribution was not retained, the rerun documents the search process but does not claim exact reconstruction of the original Scopus contribution.

Eligibility and screening. Eligible records concerned adult human studies, reviews, consensus statements, or methodological reports addressing at least one relevant behavioral exposure—eating timing or regularity, activity timing or fragmentation, sleep regularity or circadian misalignment, or free-living digital phenotyping—and a vascular, glycemic, cardiometabolic, or relevant measurement outcome. Animal-only or pediatric-only reports, pharmacological chronotherapy as the main focus, limited conference abstracts, device-engineering reports without human application, and clearly out-of-scope studies were excluded during broad screening. Of 1081 unique records, 694 proceeded to strict screening. The strict screen required both a relevant behavioral-timing exposure and a primary vascular or glycemic outcome; 257 core candidate records were retained. A targeted athlete-focused search contributed 46 records, producing a 303-record candidate evidence pool for topic mapping and prioritization. From this pool, 131 high-priority records were taken forward for full-text narrative synthesis. Because a narrative review does not require every synthesized record to appear as a direct citation, the final manuscript cites 92 sources that most directly support its focused conceptual, mechanistic, measurement, vascular, glycemic, and athlete-specific arguments. The remaining high-priority records informed interpretation and contextual comparison but were not cited separately because of scope and length constraints and to avoid redundant citation of substantively overlapping evidence. Thus, 303 denotes the candidate evidence pool, 131 denotes records assessed and used at full-text synthesis level, and 92 denotes sources directly cited in the manuscript.

Data charting and synthesis. Candidate records were grouped by behavioral domain, outcome domain, population, and study design. Priority labels used during the working review process denoted topical relevance rather than methodological quality. High-priority records provided direct coverage of a target behavioral exposure and vascular or glycemic endpoint; medium-priority records provided indirect, mechanistic, measurement, or contextual relevance; and low-priority records provided peripheral background value. The 303-record candidate pool supported topic mapping and prioritization. Following full-text relevance assessment, all 131 high-priority records informed the narrative synthesis through evidence comparison, interpretation, and identification of convergent or heterogeneous findings. Direct citation was then limited to 92 sources that provided the clearest and most section-specific support, while substantively overlapping records were used contextually without separate citation to maintain focus and comply with manuscript-length constraints. Intervention evidence, observational associations, mechanistic evidence, reviews, and athlete-focused evidence were interpreted separately and were not assigned a common certainty rating.

Population and sex. Studies of men, women, and mixed-sex samples were eligible; sex was not an exclusion criterion. Because many source studies did not report sex-disaggregated estimates, the present review cannot establish sex-specific effects. Menstrual status, menopausal status, pregnancy, energy availability, training status, adiposity, and medication use are treated as potential contextual factors or effect modifiers where reported.

## 2. Rhythm Coherence

Conceptual status. “Rhythm coherence,” the “average-value dilemma,” and the proposed RCI are author-defined concepts used to organize testable questions. They are distinct from established constructs such as circadian phase, chronotype, social jetlag, sleep regularity, and rest–activity rhythm metrics. The proposed framework does not establish that cross-behavior alignment is causal, superior to established measures, or clinically actionable.

### 2.1. Three-Layer Rhythm Definition

For the purposes of this proposed framework, rhythm coherence is provisionally described as the alignment of three behavioral rhythm layers within the 24 h cycle. The eating rhythm layer encompasses meal timing, meal frequency, meal jitter, breakfast adherence, and late-night eating frequency [5,8,9,10,11]. Meal timing is defined as the interval between the first and last daily energy intake episodes. Meal jitter refers to day-to-day variability in meal start times. Breakfast adherence refers to the proportion of days with early-day energy intake. The activity rhythm layer includes the timing of peak MVPA, also termed the activity acrophase, as well as sedentary fragmentation, sedentary-break frequency, and circadian activity indices [6,12]. These indices include interdaily stability, intradaily variability, and relative amplitude, which quantify the regularity and consolidation of 24 h activity patterns. Sedentary fragmentation describes the number, duration, and distribution of sedentary bouts. The rest–sleep rhythm layer comprises the sleep regularity index, mid-sleep time variability, and social jetlag [13]. The sleep regularity index estimates the probability of being asleep at the same clock time across days. Social jetlag reflects differences in sleep timing between workdays and free days. These three layers are interdependent rather than isolated. They are coupled through central and peripheral circadian clocks, including the suprachiasmatic nucleus (SCN), and through behavioral interactions. Examples include the influence of meal timing on activity capacity and the influence of activity timing on sleep onset. Rhythm coherence captures this integrated view by assessing both the properties of each rhythm layer and their temporal alignment and stability as an integrated system.

### 2.2. Operational Definition of Rhythm Coherence

We provisionally define rhythm coherence as a three-part construct. First, phase locking within each rhythm layer is reflected by stable daily timing of meals, physical activity, and sleep. Second, cross-day stability is reflected by low jitter and low fragmentation across rhythm layers. Third, cross-rhythm coupling is reflected by temporal coordination among layers, particularly the alignment of meals with subsequent activity and the placement of the eating window relative to the sleep period. This definition distinguishes rhythm coherence from chronotype. Chronotype is a relatively stable trait that reflects preferred sleep–wake timing. In contrast, rhythm coherence is a day-to-day behavioral state. It reflects the alignment between an individual’s real-world schedule and biological timing, as well as the consistency with which that schedule is maintained. Thus, two individuals with similar chronotypes may differ substantially in rhythm coherence. One individual may eat and train at consistent times while maintaining stable sleep. Another may exhibit irregular meal timing, fragmented activity, and variable sleep onset.

**Figure 1:**
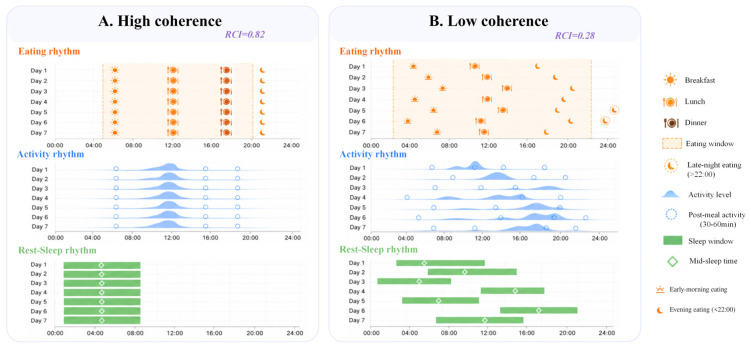
Longitudinal multimodal phenotypic tracking of high and low rhythm coherence over 7 days. The side-by-side comparison shows daily behavioral synchronization across three rhythm layers: eating (top), activity (middle), and rest–sleep (bottom), from Day 1 to Day 7. Panel (**A**) shows a high-coherence profile (RCI = 0.82), characterized by restricted eating windows, synchronized physical activity peaks consistently occurring 30–60 min after food intake, and stable rest–sleep cycles with minimal day-to-day drift in mid-sleep timing. In contrast, Panel (**B**) shows a low-coherence profile (RCI = 0.28), marked by erratic and fragmented meal timing, frequent late-night eating, physical activity peaks uncoupled from metabolic inputs, and marked temporal misalignment of sleep windows. Digitized phenotypic markers are defined as follows: sun and cutlery icons denote meals; the sunrise-over-horizon icon denotes early-morning eating; the plain crescent-moon icon denotes evening eating before 22:00; dashed orange regions indicate eating windows; blue waveforms represent continuous activity levels; dashed blue circles highlight targeted post-meal activity; solid green bars indicate sleep intervals; and open green diamonds mark mid-sleep timing. The RCI values (0.82 and 0.28) are hypothetical illustrations and are not validated thresholds or clinical categories. RCI, Rhythm Coherence Index.

Meal–activity coupling is an author-proposed cross-rhythm candidate metric [7]. To support future operationalization, we outline a candidate metric system; it has not been validated as a composite exposure or shown to outperform established single-domain measures. As detailed in Table 1, the core metrics of the Rhythm Coherence Index (RCI) are derived from three behavioral layers: eating, activity, and rest–sleep rhythms. The inclusion of meal–activity coupling as a cross-rhythm component distinguishes this framework from traditional single-modality assessments. This metric captures the temporal coordination between energy intake and energy expenditure. Specifically, it assesses whether energy intake is followed by an activity response, such as postprandial walking, within a physiologically meaningful window, typically 30–60 min. This linkage integrates nutritional timing with glucose-disposal capacity. Multimodal phenotyping studies suggest that meal–activity coupling may explain more variation in glycemic outcomes than eating-window length alone. These findings support the proposed role of meal–activity coupling within the rhythm coherence construct. Figure 1 illustrates how high and low rhythm coherence may manifest across eating, activity, and rest–sleep rhythms over a 7-day monitoring period.

### 2.3. Why Coherence Matters Beyond Single-Rhythm Metrics

Single-rhythm metrics, such as eating-window length, total daily MVPA, or sleep duration, explain only part of the inter-individual variance in cardiometabolic outcomes. This explanatory gap may be partly addressed by cross-rhythm alignment, defined as the temporal coordination among eating, physical activity, and sleep. Digital phenotyping studies illustrate this principle. Individuals with similar eating-window lengths but different meal–activity coupling patterns may show markedly different glycemic-variability profiles [19]. This observation suggests that combinations of behavioral rhythms may be more informative than any single metric. Similarly, time-restricted eating (TRE) trials that fix eating-window length without controlling meal timing relative to physical activity may show attenuated glycemic benefits compared with protocols that align the eating window with the biological morning [17,18,20]. Compositional analyses of 24 h activity patterns indicate that the daily distribution of sedentary time, light physical activity, and MVPA may predict arterial stiffness more effectively than total MVPA minutes alone [22,24]. This association suggests that the temporal structure of physical activity, not only its volume, may shape vascular adaptation.

This cross-rhythm perspective has an important practical implication. Testing the proposed rhythm-coherence construct would require multimodal digital phenotyping. A single wearable device, such as an actigraphy monitor, can capture activity and sleep layers but not the eating layer. Conversely, CGM captures glycemic dynamics but not their behavioral drivers. Cross-rhythm coupling metrics can be computed only when all three layers are measured simultaneously. Eating timing can be assessed through meal logging or CGM-derived glucose-rise patterns, activity timing through actigraphy, and sleep timing through actigraphy or polysomnography. This measurement requirement motivates the multimodal phenotyping framework developed in Section 4. This perspective also reframes intervention design. Rather than targeting a single behavior in isolation, rhythm coherence interventions should address relationships among behaviors. These interventions may aim to ensure that meals are followed by physical activity, that activity timing supports rather than disrupts sleep onset, and that the eating window is preferentially placed within the biological morning rather than the circadian night. This systems-level framing provides the conceptual foundation for the just-in-time adaptive interventions discussed in Section 7. It also distinguishes the rhythm coherence approach from existing single-modality chrononutrition and exercise-timing interventions.

## 3. Mechanisms

### 3.1. Central–Peripheral Clocks and Zeitgebers

The SCN functions as the master circadian pacemaker and synchronizes peripheral clocks in metabolic tissues, including the liver, adipose tissue, skeletal muscle, and vasculature. These peripheral oscillators are driven by transcription–translation feedback loops involving core clock genes. CLOCK and BMAL1 constitute the positive arm, whereas PER1/2/3 and CRY1/2 constitute the negative arm. Together, these loops generate approximately 24 h oscillations in gene expression and regulate the temporal organization of metabolic processes. Although peripheral clocks maintain intrinsic periodicity, they require entrainment signals, or zeitgebers, to remain phase-aligned with the SCN and with one another. Food intake and physical activity are two major zeitgebers that regulate peripheral clock timing in metabolic tissues. Nutrient-sensing pathways couple meal timing to hepatic and adipose clock phase [14,15]. For example, sirtuin 1 (SIRT1), an NAD^+^-dependent deacetylase, deacetylates BMAL1 in response to fasting-associated increases in NAD^+^. AMP-activated protein kinase (AMPK) phosphorylates CRY1 and accelerates its degradation. These processes may help reset the hepatic clock in response to energy-intake timing. The mechanistic target of rapamycin (mTOR) pathway integrates nutrient availability with clock-gene translation, thereby linking postprandial anabolic signaling to peripheral oscillator phase.

Exercise also entrains the skeletal muscle clock through parallel signaling pathways [28]. These pathways include activation of peroxisome proliferator-activated receptor gamma coactivator 1-alpha (PGC-1α), a transcriptional coactivator that interacts with BMAL1; AMPK-mediated phosphorylation of clock components; and exercise-induced increases in core body temperature, which may act as thermogenic zeitgebers for peripheral tissues. When food intake or exercise occurs at circadian phases misaligned with the SCN, peripheral clocks may become uncoupled from central pacemaker control. Examples include late-night eating and evening-only activity in individuals with a morning chronotype. This uncoupling can create a state of internal circadian desynchrony [8,9]. For example, the liver clock may become entrained to a late-night feeding schedule while the SCN remains entrained to the light–dark cycle. This pattern may produce a 4–8 h phase difference between central and peripheral oscillators. This internal desynchrony resembles the chronic circadian disruption experienced by shift workers and may contribute to the cardiometabolic consequences of rhythm incoherence. Exercise timing adds a second regulatory layer. Morning exercise may reinforce SCN–peripheral clock alignment by providing a zeitgeber that is phase-concordant with the light signal [29]. In contrast, evening exercise may delay peripheral clock phase. When performed close to bedtime, it may suppress melatonin onset and fragment sleep, thereby compounding rhythm disruption.

### 3.2. Rhythm Misalignment, Endothelial Dysfunction, and Arterial Stiffness

Endothelial dysfunction is an important early step in atherosclerosis and arterial stiffening. Endothelial nitric oxide synthase (eNOS) exhibits a pronounced diurnal rhythm, with peak expression and activity in the morning. This rhythm is influenced by clock-controlled transcription of the NOS3 gene and by morning increases in shear stress associated with activity-related elevations in cardiac output. Late-night eating and circadian misalignment may disrupt this rhythm through two parallel mechanisms. First, postprandial lipemia and hyperglycemia during the circadian night may increase reactive oxygen species (ROS). ROS can scavenge nitric oxide (NO), impair flow-mediated dilation (FMD), and increase endothelial adhesion molecules, including intercellular adhesion molecule 1 (ICAM-1), E-selectin, and vascular cell adhesion molecule 1 (VCAM-1). Second, sympathetic nervous system activation associated with late-night energy intake may increase 24 h blood pressure and arterial stiffness, commonly assessed by pulse wave velocity (PWV) [6]. This response may be mediated partly by the orexin system and the hypothalamic–pituitary–adrenal axis, thereby compounding endothelial stress.

At the systemic level, chronic circadian misalignment may promote structural vascular changes that extend beyond acute endothelial dysfunction. Shift workers often experience chronic misalignment between behavioral schedules and the light–dark cycle. They may exhibit elevated PWV, increased carotid intima-media thickness (cIMT), and attenuation of the normal nocturnal blood pressure dip [16,30]. These features are consistent with accelerated vascular aging. These structural changes may reflect cumulative exposure to repeated endothelial stress, compounded by chronic sympathetic dominance and low-grade inflammation associated with sustained rhythm incoherence. The mechanistic relationship may be bidirectional. Misaligned eating and activity rhythms may disrupt the peripheral vascular clock. This disruption may impair endothelial NO production and vasodilatory tone, thereby creating a self-reinforcing cycle of vascular dysfunction. It is important to distinguish acute postprandial endothelial effects, referred to here as T1 effects, from chronic structural changes, referred to here as T2/T3 effects [31,32]. T1 effects typically resolve within 2–4 h, whereas T2/T3 effects may accumulate over weeks to months of sustained rhythm misalignment. Acute endothelial dysfunction may represent the mechanistic entry point, whereas chronic vascular remodeling may represent the clinically relevant outcome. Rhythm coherence interventions may attenuate chronic structural vascular changes by reducing the frequency and magnitude of acute endothelial stress. Potential strategies include aligning meals with the circadian morning, coupling meals with postprandial activity, and preserving the nocturnal fasting window [33,34].

### 3.3. Rhythm Misalignment and Diurnal Insulin Sensitivity

Insulin secretion and hepatic glucose output exhibit robust diurnal rhythms. β-cell responsiveness and insulin sensitivity peak in the morning and progressively decline toward the evening. This diurnal pattern is regulated by clock-controlled expression of key molecular regulators. BMAL1 contributes to transcriptional control of the insulin gene promoter in β-cells. PER2 modulates glucocorticoid receptor sensitivity in hepatocytes and may gate the morning suppression of hepatic glucose output. These mechanisms support a physiological “metabolic morning window.” During this window, a given energy load may produce lower postprandial glucose, lower insulin demand, and faster glucose clearance than the same load consumed in the evening [13,21]. Isocaloric meal studies provide some of the clearest human evidence for this diurnal effect. When matched macronutrient loads are consumed at breakfast and dinner, evening meals may produce postprandial glucose peaks that are 20–30% higher and insulin responses that are 40–50% greater, despite equivalent energy content [35]. This time-of-day effect is not fully explained by differences in physical activity or sleep between conditions. Rather, it appears to reflect intrinsic diurnal variation in β-cell function and peripheral insulin sensitivity. Large-breakfast interventions in individuals with type 2 diabetes suggest that shifting energy intake toward the morning may reduce glycated hemoglobin (HbA1c), fasting glucose, and postprandial glucose variability over 12 weeks [36]. However, claims that such interventions are comparable to pharmacological therapies should be interpreted cautiously and supported by direct comparative evidence.

Meal–activity coupling may amplify diurnal insulin sensitivity through a partly insulin-independent mechanism. MVPA performed within 30–60 min after eating may enhance glucose clearance through glucose transporter type 4 (GLUT4) translocation to the skeletal muscle cell membrane [37]. This response is mediated partly by AMPK activation and calcium signaling induced by muscle contraction. Because this pathway can operate partly independently of insulin receptor signaling, postprandial activity may improve glycemia even in individuals with insulin resistance. Conversely, late-night eating without subsequent activity may create a “double-hit” exposure. This exposure combines an unfavorable circadian phase, characterized by lower β-cell responsiveness and higher hepatic glucose output, with a behavioral context lacking contraction-mediated, GLUT4-dependent glucose clearance [38,39]. Continuous glucose monitoring studies indicate that individuals with high meal jitter and late-night eating windows may exhibit elevated glycemic variability and reduced time in range, even after adjustment for total daily energy intake [40,41]. These findings support meal-timing regularity as a potential independent determinant of glycemic health.

### 3.4. Common Downstream Pathways

The vascular and glycemic pathways outlined above converge on three shared downstream mechanisms: systemic inflammation, oxidative stress, and autonomic nervous system imbalance. Each mechanism has its own diurnal rhythm, which may be disrupted by circadian misalignment. Each may also impair both vascular and glycemic function [42].

Systemic inflammation follows a diurnal pattern. Under normal entrainment, circulating C-reactive protein (CRP), interleukin 6 (IL-6), and tumor necrosis factor alpha (TNF-α) often peak in the early morning. Circadian misalignment, whether related to shift work, late-night eating, or irregular sleep, may blunt this morning peak and elevate baseline inflammatory tone across the 24 h cycle [43,44]. Chronically elevated IL-6 may impair insulin receptor signaling in skeletal muscle and adipose tissue, thereby contributing to insulin resistance. IL-6 may also promote endothelial ICAM-1 expression and smooth muscle cell proliferation, thereby contributing to arterial stiffening. TNF-α may suppress eNOS expression and promote endothelial apoptosis, thereby linking inflammatory signaling to vascular dysfunction.

Circadian disruption may amplify oxidative stress through two mechanisms. The first mechanism is increased mitochondrial ROS production during metabolically active periods outside the usual circadian window. One example is late-night digestion, when antioxidant enzyme expression may be at its nadir. The second mechanism is reduced expression of clock-controlled antioxidant genes during misaligned phases, including nuclear factor erythroid 2-related factor 2 (NRF2) target genes and superoxide dismutase [45,46]. ROS can scavenge NO in the endothelium, impair FMD, and oxidize low-density lipoprotein (LDL) particles, thereby accelerating atherogenesis. In the glycemic pathway, ROS may impair GLUT4 translocation and insulin receptor tyrosine kinase activity, thereby reducing glucose uptake capacity.

Autonomic imbalance, characterized by reduced parasympathetic tone and elevated sympathetic activity, may be both a cause and a consequence of rhythm incoherence. Late-night eating may activate the sympathetic nervous system through orexin signaling and postprandial thermogenesis. This activation may increase heart rate and blood pressure during a period when parasympathetic dominance would normally prepare the cardiovascular system for sleep. Reduced heart rate variability (HRV), a marker of parasympathetic withdrawal, has been observed in individuals with high meal jitter, late-night eating, and fragmented sleep [47]. Sympathetic dominance may raise fasting glucose by stimulating hepatic glucose output. It may also impair insulin secretion through α-adrenergic inhibition of β-cells. These mechanisms provide a plausible autonomic link between rhythm incoherence and glycemic dysregulation.

These three pathways (inflammation, oxidative stress, and autonomic imbalance) are interdependent and may form a mutually reinforcing triad. Inflammation may promote oxidative stress. Oxidative stress may impair autonomic regulation through effects on baroreceptors and cardiac autonomic neurons. Autonomic imbalance may amplify inflammatory responses through neuroimmune crosstalk. This mechanistic convergence suggests that rhythm coherence interventions targeting one pathway may produce downstream benefits in the other two. It also supports the hypothesis that vascular and glycemic outcomes may improve concurrently in response to coherence-restoring behavioral changes.

## 4. Multimodal Digital Phenotyping

### 4.1. Single-Modality Inventory

Although single-modality devices provide detailed insights into isolated behaviors, future evaluation of the proposed RCI would require a multimodal phenotyping stack. Table 2 outlines the measurement framework by mapping signal layers, including eating, activity, and rest–sleep rhythms, to specific modalities, such as digital meal logging and accelerometry, and to their corresponding core metrics. Table 2 also highlights a clinical outcome layer that links behavioral exposures to vascular readouts, such as ambulatory blood pressure monitoring (ABPM), and metabolic readouts derived from CGM. This integration is essential for capturing the joint effects of behavioral timing exposures on cardiometabolic health.

(i) Continuous Glucose Monitoring (CGM): CGM devices sample interstitial glucose every 1–5 min over 7–14 days and provide metrics including TIR (percentage of time at 70–180 mg/dL), CV, MAGE, and AUC. Within the rhythm coherence framework, CGM provides a metabolic readout of meal–activity coupling because postprandial glucose trajectories reflect both meal timing and subsequent activity responses [25,57]. A high-carbohydrate meal consumed at 21:00 may produce a larger and more prolonged glucose excursion than the same meal consumed at 12:00, even when total energy content is matched. This difference may reflect circadian rhythms in hepatic glucose uptake and peripheral insulin sensitivity, which generally peak in the morning [58,59]. When explicit meal logs are unavailable, the first glucose rise of the day may serve as an indirect marker of eating-window onset. This approach may enable passive estimation of eating rhythms from CGM data alone [60,61]. A critical caveat applies to non-diabetic populations. Factory-calibrated sensors may show mean absolute relative differences of 15–25% compared with venous reference measurements in normoglycemic adults, which may inflate apparent variability [62,63]. Therefore, relative within-person comparisons and directional changes may be more informative than absolute glucose values when sensor precision is limited.
(ii) Actigraphy: Wrist- and hip-worn accelerometers used continuously for seven days or longer provide information on the activity and sleep rhythm layers. Beyond total MVPA, actigraphy provides circadian activity indices. These include interdaily stability (IS; range: 0–1, with higher values indicating greater day-to-day regularity), intradaily variability (IV; range: 0–3, with higher values indicating greater within-day fragmentation), and relative amplitude (RA; range: 0–1, with higher values indicating greater active–rest contrast). These indices characterize the structure of 24 h activity patterns and may predict cardiometabolic outcomes independently of MVPA volume [6,23,24]. For example, an individual who accumulates 150 min/week of MVPA in irregular, fragmented bouts would be expected to have lower IS and higher IV than an individual with the same activity volume distributed across consistent daily patterns. The irregular pattern may be associated with higher PWV and poorer glycemic control. MVPA acrophase quantifies activity timing and enables calculation of the phase-alignment index, defined as the absolute difference between meal and activity acrophases. The SRI and mid-sleep time variability can be derived from the same actigraphy data stream without separate sleep diaries. Thus, actigraphy is one of the most data-rich single modalities in the multimodal phenotyping stack [53,54].
(iii) Digital Meal Logging: Digital meal logging captures the eating rhythm layer, which CGM and actigraphy cannot directly measure. Methods include structured 24 h dietary recalls, such as the Automated Self-Administered 24-Hour Dietary Assessment Tool (ASA24), smartphone-based ecological momentary assessment (EMA), image-based artificial intelligence (AI) logging, and passive acoustic sensing with throat-worn microphones to detect chewing events. For rhythm coherence research, key outputs include eating-window start and end times, meal jitter, eating frequency, macronutrient timing, and the proportion of total daily energy intake consumed after 20:00 [48,49,50]. Meal jitter can be operationalized as the standard deviation of meal start times across days and expressed in minutes. The primary limitation is declining adherence over time. Real-time logging adherence often decreases after days 3–5, and underreporting of 20–30% is common even among motivated participants [51,52]. Image-based AI logging may reduce participant burden but can introduce classification errors for mixed dishes and culturally specific foods. Passive acoustic sensing avoids active logging but cannot capture macronutrient composition. For studies requiring at least 14 days of eating-rhythm data, a hybrid approach may provide the best balance between data completeness and participant burden. Such an approach could combine structured recalls for macronutrient composition with passive, CGM-derived estimation of the eating window.
(iv) Ambulatory and Cuffless Blood Pressure Monitoring: ABPM remains the reference standard for assessing the 24 h blood pressure (BP) profile, including nocturnal dipping status, morning surge, and BP variability across the diurnal cycle. Validated ABPM devices typically inflate every 15–30 min over 24 h and provide 48–96 readings to characterize the diurnal BP pattern. Key derived metrics for rhythm coherence research include the nocturnal dipping ratio, defined as the night/day mean BP ratio; morning surge magnitude, defined as the difference between the post-waking peak and pre-waking trough; and 24 h BP variability, estimated using average real variability or standard deviation [64,74]. Non-dipping, defined as a nocturnal BP decline of less than 10%, and reverse dipping are independent predictors of cardiovascular events. They may also be mechanistically linked to rhythm misalignment through sympathetic activation during the sleep period [55,56]. This pathway may help explain how late-night eating and irregular sleep timing contribute to elevated nocturnal BP. Cuffless BP wearables, many of which are based on optical photoplethysmography (PPG), enable continuous monitoring without cuff-inflation discomfort. However, clinical validation against ABPM remains incomplete. Therefore, within-person longitudinal tracking of directional BP changes may currently be the most appropriate application of cuffless devices in rhythm coherence research [7].
(v) Flexible Multimodal Biosensors: Flexible epidermal biosensors extend digital phenotyping beyond glucose and movement to include biochemical and autonomic dimensions of rhythm coherence. Thin-film electrochemical patches can simultaneously measure sweat electrolytes, including sodium (Na^+^) and potassium (K^+^); metabolic substrates, including lactate and glucose; and cortisol with high temporal resolution during exercise and daily activity. Electromyography (EMG) patches and skin-temperature sensors provide proxies for muscle activation and autonomic tone. When integrated with CGM and actigraphy, these sensors may capture biochemical correlates of rhythm misalignment that are not detectable using glucose and movement sensors alone. Examples include elevated cortisol during late-night eating and lactate accumulation after poorly timed high-intensity training.

### 4.2. Multimodal Integration Taxonomy

Integrating continuous glucose monitoring (CGM), actigraphy, meal logging, and ambulatory blood pressure monitoring (ABPM) requires three levels of multimodal fusion [7]. Level 1, data fusion, aligns raw signals on a common time axis: CGM at 5 min epochs, actigraphy at 30 s epochs, meal logs at the event level, and ABPM at 15–30 min intervals. Device clock drift, time-zone changes during travel, and sensor removal during water-based activities can introduce data gaps. These gaps require predefined imputation or exclusion rules before cross-modal analysis.

Level 2, feature fusion, combines derived metrics and cross-rhythm indices. For example, phase alignment requires meal acrophase derived from meal logs and activity acrophase derived from actigraphy. In contrast, meal–activity coupling requires matching meal timestamps to subsequent activity bouts in the actigraphy stream.

**Figure 2:**
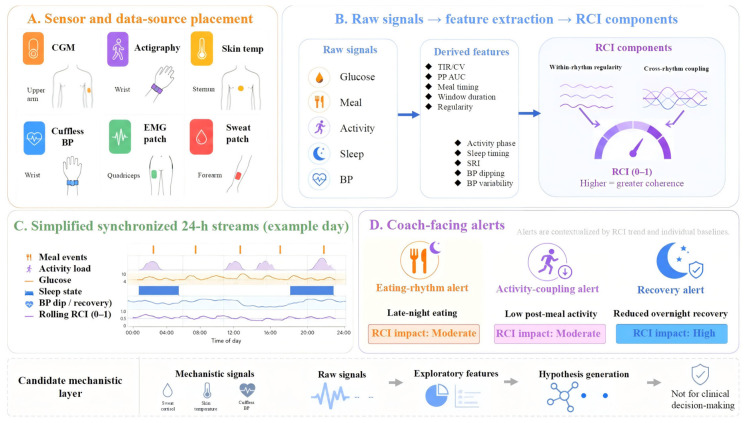
Hypothesis-generating example of a possible multimodal digital phenotyping architecture for future evaluation of the Rhythm Coherence Framework. The four panels are conceptual design examples rather than a validated end-to-end system. Panel (**A**) illustrates possible anatomical placement of six candidate sensors: continuous glucose monitoring (CGM) on the upper arm; actigraphy and cuffless blood pressure (BP) on the wrist; skin-temperature sensing on the sternum; an electromyography (EMG) patch on the quadriceps; and a sweat patch on the forearm. Panel (**B**) illustrates a possible signal-processing workflow. Raw multisensor streams could undergo feature extraction to generate candidate metrics, including time in range (TIR), coefficient of variation (CV), postprandial area under the curve (PP AUC), and the Sleep Regularity Index (SRI). These examples show how a future candidate RCI might combine within-rhythm regularity and cross-rhythm coupling; the displayed 0–1 range, components, and workflow are hypothetical and unvalidated. Panel (**C**) provides a simplified synthetic 24 h data-stream example rather than participant data. Panel (**D**) is a speculative interface mock-up showing possible alerts for eating rhythm, activity coupling, and recovery. Neither the alerts nor their displayed RCI impacts have been derived or tested. The candidate mechanistic layer is similarly exploratory. All pathways, RCI values, alerts, and decision-support elements require prospective development and validation and must not be interpreted as available algorithms, clinical thresholds, coaching tools, or treatment recommendations. In Panel (**C**), the thick blue horizontal segments indicate illustrative sleep-state intervals. In Panel (**D**), the composite icons respectively denote late-night eating, low post-meal activity, and reduced overnight recovery; they are illustrative alert symbols rather than validated clinical indicators. Colors are used as illustrative visual cues to distinguish data streams and alert categories and do not represent validated clinical risk levels. AUC, area under the curve; BP, blood pressure; CGM, continuous glucose monitoring; CV, coefficient of variation; EMG, electromyography; PP AUC, postprandial area under the curve; RCI, Rhythm Coherence Index; SRI, Sleep Regularity Index; TIR, time in range.

Level 3, decision fusion, represents a hypothetical future stage in which modality-specific features could be evaluated in composite research models. Future studies could compare prespecified composite approaches with machine-learning classifiers trained on multimodal feature vectors. No RCI weighting or decision rule has been validated. At least seven valid monitoring days may be a reasonable starting point for methodological evaluation to capture weekly behavioral patterns, including the workday–free-day contrast that defines social jetlag. Studies designed to detect seasonal variation or menstrual-cycle effects on rhythm coherence require proportionally longer monitoring windows. Figure 2 presents a hypothesis-generating design scenario, not an implemented or validated digital-health system.

### 4.3. Quantifying Rhythm Coherence

Within-rhythm metrics that could be evaluated include eating-window length, meal jitter, breakfast adherence, late-night eating frequency, activity acrophase, interdaily stability, intradaily variability, relative amplitude, sedentary fragmentation, the Sleep Regularity Index, and mid-sleep variability. Candidate cross-rhythm measures include the proportion of meals followed by activity within a prespecified interval and the phase difference between eating and activity patterns. These examples are proposed measurement options rather than validated RCI components. Operational definitions, monitoring duration, directionality, and preprocessing rules would need to be prespecified and tested across devices and populations.

A future RCI might combine standardized within-rhythm and cross-rhythm components, but a definitive equation is not proposed here. Component selection, scaling, weighting, interaction terms, missing-data rules, minimum wear time, and the handling of collinearity remain unresolved. Equal weighting should not be treated as a default clinical solution. Development should begin with content and construct validity, followed by reliability, criterion validity against prespecified vascular and glycemic outcomes, responsiveness, calibration, discrimination, and external validation. Thresholds would require independent derivation and validation in relevant populations before any risk classification or decision-support use. Activity-composition variables should be analyzed with methods that respect the 1440 min constraint, such as appropriate log-ratio approaches [6]. Candidate considerations for future RCI development are summarized in Box 1.

Box 1 **Candidate Metric System for the Rhythm Coherence Index (RCI).**

No validated RCI equation or scoring rule currently exists.

Any future candidate metric system would require prespecified component definitions, directionality, scaling, weights, interaction terms, and missing-data rules. These decisions should be evaluated for reliability, validity, responsiveness, calibration, and transportability before a summary score or threshold is interpreted.

### 4.4. Behavioral Rhythm Phenotypes

Beyond continuous RCI scores, multimodal data may support the clustering of individuals into discrete behavioral rhythm phenotypes that capture nonlinear cardiometabolic risk patterns. An ecological momentary assessment (EMA)-based study identified five overeating phenotypes that differed in meal-timing regularity, late-night eating frequency, and meal–activity coupling [19]. Phenotype membership was associated with glycemic variability beyond eating-window length alone [67,75]. Multimodal studies in Hispanic/Latino adults have identified meal–activity coupling as a strong cross-rhythm correlate of glycemic variability, with greater explanatory value than any single behavioral modality [7]. These phenotypes may provide candidate behavioral exposure units for the evidence synthesis in Section 5.

## 5. Rhythm Coherence and Cardiometabolic Outcomes

Evidence-status note. The studies summarized in this section differ substantially in design, population, exposure definition, and outcome assessment. Mechanistic plausibility does not establish clinical benefit; observational associations do not establish causality; and effects from time-restricted eating, exercise-timing, sleep, or postprandial-activity interventions cannot be attributed to an integrated RCI that was not measured. Quantitative estimates are therefore interpreted only in the context of the source design and intervention.

### 5.1. Vascular Backbone

Arterial stiffness, commonly quantified using PWV and the augmentation index (AIx), is a robust predictor of cardiovascular events and mortality. It reflects cumulative structural and functional changes in the arterial wall, including increased collagen cross-linking, reduced elastin content, and vascular smooth muscle hypertrophy. Observational studies in shift workers, a population model of chronic circadian misalignment, have reported higher PWV than in day workers. This association persisted after adjustment for traditional risk factors, including age, body mass index, blood pressure, and smoking [12]. Cross-sectional studies have also linked social jetlag, defined as a mismatch between biological and social sleep timing, to higher PWV. This finding suggests that chronotype–behavior misalignment may represent a modifiable risk factor independent of total sleep duration. The reported effect size may be clinically meaningful. Each additional hour of social jetlag has been associated with approximately 0.3–0.5 m/s higher PWV [6], a magnitude that may correspond to several years of vascular aging.

Recent compositional analyses using Aitchison log-ratio methods suggest that reallocating 30 min of sedentary time to moderate-to-vigorous physical activity (MVPA) is associated with lower PWV [22,23]. This association may be stronger when activity is temporally aligned with eating patterns rather than concentrated in a single daily bout. Selected time-restricted-eating and exercise studies have reported reductions in systolic blood pressure or improvements in vascular measures in adults with overweight or cardiometabolic risk [68]. These intervention-specific findings should not be interpreted as an effect of rhythm coherence or the proposed RCI. The proposed mechanistic pathway may involve both acute and chronic processes. Acute effects may include improved post-exercise endothelial function and reduced postprandial sympathetic activation. Chronic adaptations may include reduced arterial wall inflammation and improved arterial compliance through nitric oxide-mediated vascular smooth muscle relaxation. The timing of activity relative to meals appears to modulate the magnitude of vascular benefit. Postprandial activity may attenuate postprandial endothelial stress and provide vascular benefits beyond those attributable to activity volume alone.

However, cross-sectional associations should be distinguished from intervention evidence. Cross-sectional studies indicate that rhythm misalignment correlates with higher PWV, whereas intervention studies are needed to determine whether improving rhythm coherence directly reduces PWV. At present, causal evidence is derived mainly from TRE and exercise-timing randomized controlled trials with surrogate vascular endpoints. Brachial artery FMD is a functional measure of endothelial nitric oxide bioavailability and predicts future cardiovascular events. Postprandial FMD impairment refers to a transient reduction in endothelial function after high-fat or high-glycemic meals. This impairment may be exacerbated by evening meal consumption, when eNOS expression may be lower [11,13]. Morning exercise may produce greater improvements in FMD than evening exercise [29]. This difference may reflect circadian phase-dependent effects on endothelial function and the acute anti-inflammatory effects of morning activity. Individuals with late-night eating patterns may also show reduced morning FMD. This pattern suggests that evening meal timing could disrupt overnight endothelial recovery [21,25]. Meal–activity coupling, defined as meal consumption followed by brief bouts of physical activity, may attenuate postprandial FMD impairment. This finding suggests that behavioral rhythm coherence could partially offset the adverse vascular effects of circadian phase mismatch. Collectively, these findings suggest that FMD may be a sensitive marker of rhythm coherence status and a useful biomarker of intervention response.

The normal 24 h blood pressure profile includes a 10–20% nocturnal dip, reflecting the transition from sympathetic to parasympathetic dominance during sleep. Loss of this dipping pattern, expressed as non-dipping or reverse-dipping, independently predicts cardiovascular events and target-organ damage. Late-night eating may disrupt nocturnal blood pressure dipping by extending postprandial sympathetic activation into the sleep period and attenuating the normal parasympathetic rebound [8]. Conversely, TRE windows that end 2–3 h before bedtime may help preserve nocturnal dipping and reduce 24 h blood pressure variability. Exercise timing may also influence the 24 h blood pressure profile. Morning-timed exercise may enhance nocturnal dipping, whereas evening exercise may blunt this response if performed too close to bedtime. Meal–activity coupling, particularly postprandial activity in the afternoon, may improve the 24 h blood pressure profile by enhancing daytime blood pressure control while preserving nocturnal dipping. Ambulatory blood pressure monitoring studies in individuals with high meal jitter have reported elevated 24 h blood pressure variability and reduced dipping. These findings suggest that eating rhythm regularity may contribute to blood pressure control independently of average intake.

### 5.2. Glycemic Outcomes

CGM enables real-time assessment of glycemic variability under free-living conditions. It has revealed associations between rhythm coherence and glucose control that are not fully captured by mean glucose levels. TRE interventions, particularly those aligned with chronotype or combined with morning exercise, have been reported to increase TIR and reduce the coefficient of variation (CV%) in both diabetic and non-diabetic populations [40,41,76]. Meal jitter, defined as day-to-day variability in eating times, is independently associated with elevated glycemic variability [17,40,65], even when total daily energy intake is held constant. Breakfast skipping or delayed breakfast consumption may lead to exaggerated postprandial glucose responses to subsequent meals, a phenomenon termed the “second-meal effect.” This phenomenon suggests that eating rhythm regularity may prime the metabolic system for efficient glucose handling. Late-night eating may disrupt the overnight fasting period and elevate next-morning fasting glucose. This disruption may increase glycemic variability throughout the following day. Multimodal phenotyping studies that combine CGM with actigraphy and meal logging further suggest that meal–activity coupling is a strong behavioral predictor of glycemic variability and may explain more variance than any single modality alone. Together, these findings provide a rationale for studying eating regularity, meal–activity timing, and sleep–wake consistency jointly, but they do not establish the proposed integrated construct as an independent determinant of glucose control.

Postprandial physical activity is one of the most effective behavioral strategies for reducing postprandial glucose excursions. A single 10 min bout of moderate-to-vigorous activity within 30 min after eating can reduce the postprandial glucose peak by approximately 20–30% and the glucose area under the curve by 15–25% [26,27]. The timing of activity relative to meal consumption also appears to matter. Activity initiated within 15–30 min after a meal appears more effective than activity delayed until 60–90 min postprandially. This timing effect likely reflects the window of maximal GLUT4 translocation and skeletal muscle glucose uptake. Exercise snacks, such as three 10 min bouts of activity distributed throughout the day, may produce glycemic benefits comparable to those of a single continuous 30 min bout. This finding suggests that meal–activity coupling, rather than total activity duration alone, may be a key determinant of postprandial glucose regulation. The dose–response relationship also appears to be non-linear. Moderate-intensity activity, approximately 50–70% VO_2_max, may be more effective than low-intensity walking for reducing postprandial glucose. In contrast, very high-intensity activity above 85% VO_2_max may provide no additional glycemic benefit. These findings support meal–activity coupling as a core component of rhythm coherence. They also provide a mechanistic rationale for why interventions combining TRE with structured postprandial activity may produce greater glycemic benefits than either strategy alone.

### 5.3. Convergence of Vascular and Glycemic Outcomes

Converging evidence across multiple domains supports the hypothesis that rhythm coherence may concurrently influence vascular and glycemic health. Table 3 summarizes the primary outcomes and overall patterns linking 24 h behavioral rhythms with vascular and glycemic endpoints. Supplementary Table S1 provides a more detailed evidence map. For example, interventions targeting eating-window optimization and time-restricted eating have generally shown favorable cardiometabolic effects, including reduced blood pressure and improved TIR. Evidence on meal-timing variability shows a complementary pattern: greater meal-timing jitter is associated with higher glycemic variability and less favorable vascular markers, including PWV. Together, these findings suggest that behavioral rhythm disruption may affect vascular and glycemic regulation through partially shared mechanisms.

The vascular and glycemic evidence streams converge on an important hypothesis: rhythm coherence may function as an integrated cardiometabolic risk construct that links behavioral timing to both vascular and glycemic endpoints. Multimodal phenotyping studies that combine CGM, actigraphy, and meal logging suggest that individuals with higher rhythm coherence may have lower arterial stiffness and better glucose control than those with fragmented rhythms. Higher coherence is characterized by regular eating times, consistent activity patterns, and stable sleep–wake cycles. Shift work studies provide a useful population model for examining this convergence. Shift workers often show elevated PWV, reduced FMD, and increased glycemic variability within the same populations. This clustering suggests the presence of shared mechanistic drivers. TRE interventions also appear to produce parallel improvements across both outcome domains. Reductions in systolic blood pressure of approximately 3–5 mmHg may co-occur with increased TIR and reduced glycemic variability [7,68]. This dual-outcome response is biologically plausible because shared downstream pathways, including systemic inflammation, oxidative stress, and autonomic imbalance, may be responsive to rhythm coherence interventions. The convergence of findings across separate behavioral domains provides a rationale for testing, rather than assuming, whether a future composite RCI adds information beyond established measures. Rather than treating eating, activity, and sleep rhythms as separate risk factors, the RCI framework conceptualizes them as components of a single integrated behavioral phenotype that may shape cardiometabolic health.

### 5.4. Modulation Across the Physical Activity Spectrum

The association between rhythm coherence and cardiometabolic outcomes may be modified by baseline physical activity level, creating a gradient across the activity spectrum. In sedentary individuals, rhythm misalignment appears to be associated with the greatest adverse effects on vascular and glycemic endpoints. For example, shift workers and individuals with high meal jitter often show elevated PWV and greater glycemic variability [20,66]. In recreationally active individuals, defined here as those accumulating approximately 150–300 min/week of moderate activity, the dose–response relationship appears clearer. Greater rhythm coherence, reflected by regular meal timing, consistent activity timing, and stable sleep, is associated with better outcomes. Interventions targeting rhythm coherence may also produce measurable improvements in both vascular and glycemic domains [78,79].

However, in highly trained athletes, defined here as individuals undertaking more than 10 h/week of structured training, this association may become paradoxical. High training volume may disrupt eating and sleep rhythms, potentially contributing to vascular stiffening and glycemic dysregulation despite high aerobic fitness. This “athlete’s paradox” may reflect competing influences: training-related circadian disruption, including early-morning training, late-evening competition, and transmeridian travel, versus the cardiometabolic protection conferred by high fitness. Individuals with high training volume but poor rhythm coherence may show higher PWV and greater glycemic variability than recreationally active individuals with better rhythm coherence [82]. This spectrum-dependent pattern highlights rhythm coherence as a distinct risk construct. It also sets the stage for Section 6, which explores how flexible multimodal biosensors may help athletes optimize both training load and behavioral rhythm coherence.

### 5.5. Adiposity, Appetite Regulation, and Vascular Inflammation

Body mass index (BMI) should be considered when interpreting associations between behavioral timing and cardiometabolic outcomes, but BMI does not distinguish fat mass, lean mass, or visceral adiposity. Body-fat distribution may be particularly relevant because visceral adipose tissue contributes to insulin resistance and vascular inflammation. Adipose-derived signals may also connect behavioral timing with appetite and vascular biology. Leptin participates in satiety signaling and is influenced by adiposity and sleep, whereas adiponectin is generally associated with insulin sensitivity and vascular protection [83,84]. Pro-inflammatory cytokines and chemokines, including interleukin-6, tumor necrosis factor-α, and monocyte chemoattractant protein-1, may contribute to endothelial activation, reduced nitric-oxide bioavailability, and arterial dysfunction [85,86]. Appetite, satiety, meal timing, sleep loss, and energy availability may therefore interact with adiposity-related pathways. In future studies, BMI, waist circumference, body-fat percentage, visceral adiposity, dietary intake, appetite-related measures, and inflammatory biomarkers should be treated as potential confounders, mediators, effect modifiers, or outcomes according to the study design. Biomarker sampling time should be standardized because several adipokines and cytokines show diurnal variation. These factors are not proposed as validated RCI components.

## 6. Rhythm Coherence in Athletes

### 6.1. The Rhythm Coherence Paradox in Athletes

Across the physical activity spectrum, higher activity levels are generally associated with better rhythm coherence and more favorable cardiometabolic outcomes. However, this gradient may attenuate or even reverse at the elite end of the spectrum. Highly trained athletes face structural pressures that may disrupt all three rhythm layers. Early-morning training sessions may shorten the overnight fasting period and fragment sleep. Transmeridian travel and evening competition schedules may impose acute circadian misalignment. High energy demands may also promote late-night refueling, thereby shifting the eating window into the circadian night. This pattern creates a paradox: athletes may exhibit poorer rhythm coherence than recreationally active adults despite superior aerobic fitness, body composition, and resting cardiovascular function. Studies of time-restricted eating in endurance runners suggest that imposing an eating window without accounting for training timing may inadvertently reduce rhythm coherence by shifting meals into suboptimal circadian phases [69]. This paradox supports the need for athlete-specific phenotyping approaches. Such approaches should incorporate training load, competition schedules, and travel as rhythm-disrupting exposures, in addition to the behavioral rhythm metrics used in general populations.

### 6.2. Hidden Vascular Risks in Elite and Master Athletes

The assumption that athletic training uniformly protects vascular health has been challenged by emerging evidence of potential hidden vascular risk in elite and master athletes. Three patterns are particularly relevant to the rhythm coherence framework. First, an arterial stiffness paradox has been described in athletic populations. Endurance athletes often show lower resting PWV than sedentary controls, consistent with expected vascular adaptations to training. However, master athletes, defined here as individuals older than 50 years with more than 20 years of training history, may show age-related increases in PWV that parallel those observed in sedentary peers [87]. This finding suggests that the vascular protection associated with training may not persist indefinitely. Reference values for PWV and blood pressure in elite athletes may differ substantially from general population norms, thereby complicating clinical interpretation [88]. Second, hypertension in athletes may represent a distinct clinical phenotype. Isolated systolic hypertension, defined as elevated systolic blood pressure with normal diastolic blood pressure, has been frequently reported in young competitive athletes, particularly those participating in strength and power sports [89]. This pattern may reflect increased arterial stiffness related to chronic pressure overload rather than the endothelial dysfunction commonly observed in sedentary hypertension. Nevertheless, it may still carry independent cardiovascular risk. Exercise-induced hypertension, characterized by an exaggerated blood pressure response to submaximal exercise, may represent an additional risk marker that can be missed during standard clinical screening. Third, lifelong endurance athletes may have a higher burden of coronary atherosclerotic plaques than late-onset athletes who began training after age 30, despite higher cardiorespiratory fitness [90]. This counterintuitive finding is consistent with the hypothesis that cumulative exposure to exercise-induced hemodynamic stress may contribute to vascular risk. This exposure may be compounded by long-term rhythm disruption, including early-morning training, competition travel, and inadequate recovery nutrition.

### 6.3. RED-S and the Cardiometabolic Intersection

Relative Energy Deficiency in Sport (RED-S) may represent one mechanism through which rhythm incoherence contributes to vascular risk in athletes. When training-induced energy expenditure is not matched by adequate energy intake, athletes may enter a state of low energy availability (LEA). The resulting hormonal changes, including suppressed estrogen, elevated cortisol, and reduced insulin-like growth factor-1 (IGF-1), may impair endothelial function and arterial compliance. The 2023 International Olympic Committee (IOC) RED-S consensus statement recognizes cardiovascular health as a primary consequence domain, alongside the more widely recognized bone and menstrual consequences [91]. Vascular studies in athletes at risk of RED-S suggest a subclinical pattern. Female elite runners with RED-S risk markers may show FMD and PWV values comparable to those of healthy controls at rest, even when a substantial proportion meet RED-S risk criteria [70]. This finding suggests that vascular impairment may be masked by the protective effects of training until energy deficiency becomes severe or prolonged. Short-term experimental LEA, even over 5 days, has been reported to reduce forearm blood flow and resting energy expenditure [73]. This suggests that brief periods of energy restriction may produce measurable vascular effects. Within the rhythm coherence framework, RED-S can be conceptualized as a downstream manifestation of eating-rhythm disruption. Athletes who skip breakfast before early-morning training compress their eating window to accommodate training schedules, or rely on late-night refueling may be at elevated RED-S risk because their eating rhythm is misaligned with their energy-expenditure pattern [92].

### 6.4. Flexible Multimodal Biosensors in Sport

Athletes represent a promising population for deploying flexible multimodal biosensor stacks. They are often motivated to optimize performance, accustomed to wearable monitoring, and embedded in structured training environments that may support high sensor adherence [71,72]. A rhythm coherence phenotyping stack for athletes could integrate four simultaneous data streams. First, CGM could capture glycemic responses to training sessions and meal timing, thereby identifying postprandial glucose trajectories that reflect the quality of meal–activity coupling. Second, sweat-sensing patches could measure Na^+^, K^+^, lactate, and cortisol as biochemical markers of hydration status, metabolic substrate utilization, and stress–recovery balance. Third, EMG and skin-temperature sensors could provide proxies for muscle activation patterns and autonomic nervous system tone. Fourth, cuffless blood pressure monitoring could detect exercise-induced hypertension and track the 24 h blood pressure profile across training and recovery days. Synchronous multi-stream output could support future evaluation of a training-adapted candidate RCI, subject to analytical and prospective validation. Such an index could account for athlete-specific rhythm-disruption profiles, including early-morning training, competition travel, and energy-availability status, and translate these profiles into actionable coaching metrics [54]. Rather than reporting abstract circadian indices alone, such a system could flag specific behavioral misalignments. Examples include elevated cortisol at 22:00 despite scheduled sleep at 23:00, which may suggest a need to move evening training earlier, or a postprandial glucose spike after a post-training meal, which may indicate suboptimal carbohydrate timing. Such a sensor-to-decision pipeline remains hypothetical and should not be used for coach–athlete decision support until measurement validity, safety, and clinical or performance utility have been established. This translational application distinguishes the athlete-focused section of this review from the existing chrononutrition and exercise-timing literature.

## 7. Cross-Scale Framework and Research Agenda

### 7.1. Cross-Scale Integration Framework

**Figure 3:**
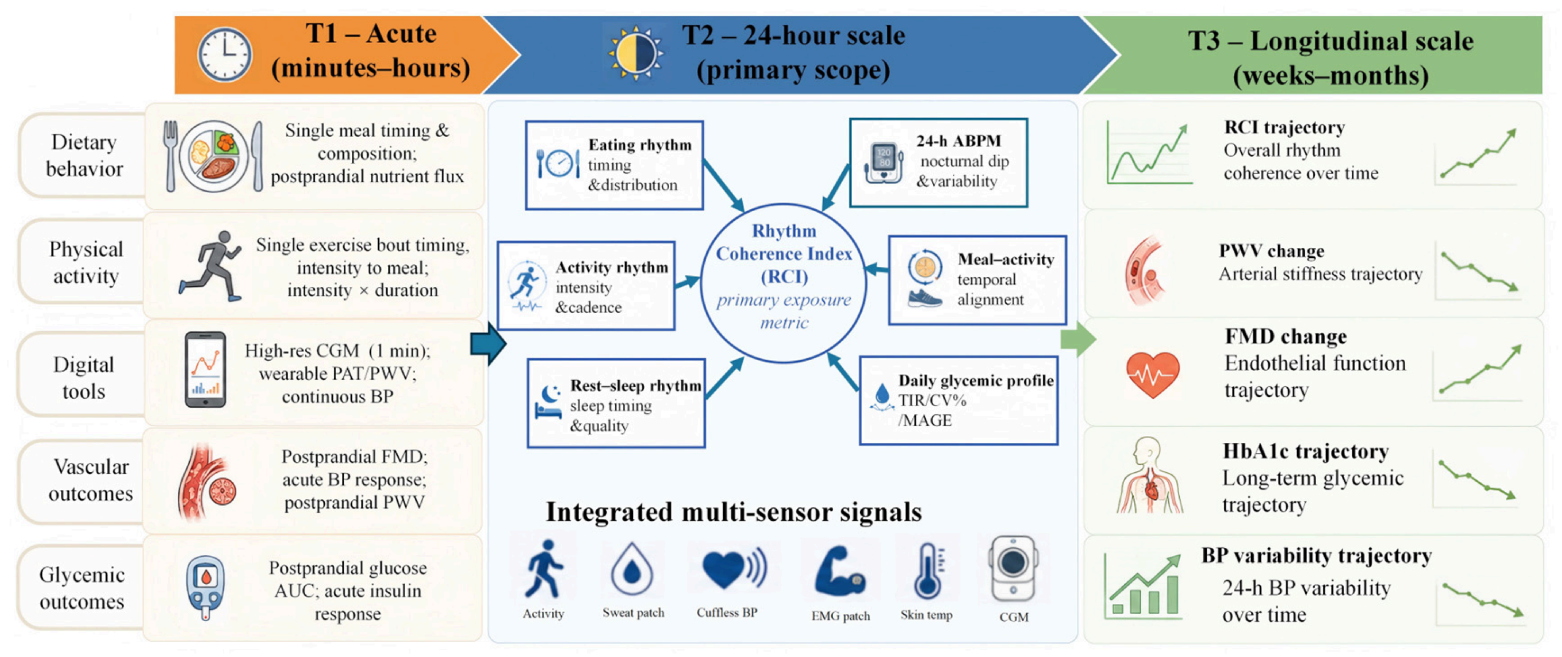
Cross-scale multimodal integration framework linking acute exposures to longitudinal cardiometabolic health trajectories through the 24 h Rhythm Coherence Index (RCI). The conceptual matrix organizes physiological, behavioral, and digital markers across five analytical dimensions: dietary behavior, physical activity, digital tools, vascular outcomes, and glycemic outcomes. These dimensions span three nested temporal scales: T1, T2, and T3. T1, the acute scale spanning minutes to hours, captures immediate postprandial nutrient flux, individual exercise bouts, and acute vascular or glycemic fluctuations. These fluctuations are detected using high-resolution digital tools, such as 1 min continuous glucose monitoring (CGM), wearable pulse amplitude tonometry (PAT), and continuous blood pressure (BP) monitoring. Thick horizontal arrows denote the temporal cascade through which acute T1 mechanisms aggregate into stabilized T2 behavioral patterns. T2 represents the 24 h scale and the primary scope of the framework. At the T2 core, integrated multisensor signals converge to operationalize the RCI through six multidimensional nodes: eating rhythm, activity rhythm, rest–sleep rhythm, 24 h ambulatory BP monitoring (ABPM), daily glycemic profiles, and meal–activity temporal alignment. These signals include activity tracking, sweat patches, cuffless BP, electromyography (EMG) patches, skin temperature, and CGM. Daily glycemic profiles include time in range (TIR), coefficient of variation (CV), and mean amplitude of glycemic excursions (MAGE). The framework hypothesizes that sustained 24 h rhythm coherence could accumulate over time and relate to T3 clinical endpoints, representing the longitudinal scale of weeks to months. Favorable longitudinal trajectories are characterized by progressive improvements in overall rhythm coherence, as reflected by the RCI trajectory, and endothelial function, as reflected by changes in flow-mediated dilation (FMD). These trajectories are also accompanied by reductions in arterial stiffness, long-term glycemia, and 24 h BP variability, as reflected by changes in pulse wave velocity (PWV), HbA1c trajectory, and BP variability, respectively. The arrows and trajectories are conceptual rather than demonstrated causal pathways, and no RCI threshold is implied. The color coding denotes the three temporal scales: orange for T1 (acute, minutes to hours), blue for T2 (24-hour scale and primary scope), and green for T3 (longitudinal, weeks to months). All icons are illustrative representations of the constructs identified by their adjacent labels and do not denote validated devices or clinical pathways. ABPM, ambulatory blood pressure monitoring; AUC, area under the curve; BP, blood pressure; CGM, continuous glucose monitoring; CV, coefficient of variation; EMG, electromyography; FMD, flow-mediated dilation; HbA1c, glycated hemoglobin; MAGE, mean amplitude of glycemic excursions; PAT, pulse amplitude tonometry; PWV, pulse wave velocity; RCI, Rhythm Coherence Index; TIR, time in range.

The rhythm coherence framework operates across three nested time scales and may help explain how acute behavioral patterns accumulate into chronic cardiometabolic risk. At the acute scale (T1; minutes to hours), the composition and timing of a single meal may influence postprandial glucose trajectories and the magnitude of transient endothelial dysfunction. The timing of a single exercise bout relative to that meal may further modulate both responses [41,65]. These acute responses can be measured using high-resolution CGM, wearable pulse-arrival-time sensors, and continuous blood pressure monitoring. At the 24 h scale (T2), which is the primary scope of this review, daily patterns of meals, activity bouts, and sleep may shape the 24 h ABPM profile, daily TIR, and glycemic variability [6,7]. T2 is the scale at which the proposed RCI could be evaluated. Such evaluation would require synchronized CGM, actigraphy, meal logging, and ABPM data collected over at least seven days. At the longitudinal scale (T3; weeks to months), habitual rhythm coherence trajectories may predict changes in PWV, HbA1c, arterial compliance, and incident hypertension [68,76]. Assessment of T3 outcomes requires periodic clinical measurements, including PWV, FMD, and ABPM, combined with long-term wearable monitoring. The value of multimodal digital phenotyping lies not in any single time scale but in its capacity to integrate information across all three. T1 mechanisms may explain how T2 behavioral patterns translate into T3 outcomes, whereas T3 outcomes provide evidence for the clinical relevance of T2 exposures. This cross-scale integration provides the conceptual architecture that distinguishes the rhythm coherence framework from single-modality approaches. Figure 3 integrates these concepts into a cross-scale framework linking acute behavioral exposures, 24 h rhythm coherence, and longitudinal cardiometabolic trajectories.

### 7.2. JITAIs and Closed-Loop Intervention Potential

Just-in-time adaptive interventions (JITAIs) deliver behavioral prompts during periods of high receptivity, using real-time sensor data to identify actionable intervention windows. CGM-triggered postprandial activity reminders may reduce postprandial glucose excursions by prompting a 10 min walk within 30 min after eating [26,27]. However, existing JITAIs have generally targeted individual behavioral domains rather than integrating all three rhythm layers into a single closed-loop behavioral system. A validated RCI might eventually be tested as one possible trigger for such systems. In a future research prototype, a prespecified change in the candidate RCI after a late meal, a missed activity bout, or delayed sleep onset, the system could deliver a prompt tailored to the most disrupted rhythm layer. For example, an eating-rhythm alert could recommend advancing the next meal, an activity-rhythm alert could suggest postprandial walking, and a recovery alert could prompt earlier sleep preparation. This closed-loop architecture may represent a translational endpoint of the rhythm coherence framework.

### 7.3. Research Priorities

(i) RCI validation. Criterion-validation studies should examine associations between the composite RCI and PWV, FMD, and TIR in well-characterized cohorts [6,7]. Candidate components and weights should be prespecified, empirically evaluated, and reported transparently; equal weighting is only one hypothesis and should not be assumed to be valid. (ii) Open, multimodal datasets. To our knowledge, no public dataset currently combines synchronized CGM, actigraphy, meal logging, and ABPM with vascular endpoints in the same participants [48,49]. An open resource with at least 500 participants, at least 14 days of wearable data, and standardized clinical assessments would accelerate cross-study replication. (iii) Stratification by sex, age, and ethnicity. Much of the existing evidence derives from predominantly White, middle-aged samples. Associations are likely to differ by menopausal status, chronotype, cultural meal patterns, and baseline cardiometabolic risk. Universal RCI thresholds therefore require stratified analyses before clinical or population-level application. (iv) Causal inference. Mendelian randomization using genetic instruments for chronotype and sleep regularity may provide causal estimates. N-of-1 wearable crossover designs could further support within-person causal inference with high ecological validity. (v) Clinical endpoints. Current evidence is largely limited to surrogate endpoints, including PWV, FMD, and TIR. Existing cohorts with accelerometry, such as UK Biobank and the Multi-Ethnic Study of Atherosclerosis, should be leveraged to examine incident cardiovascular disease and type 2 diabetes. (vi) Athlete norms and flexible biosensor validation. Sport-specific RCI reference values are currently lacking. Sweat-based biomarkers, including cortisol and lactate, require validation against circadian and biochemical gold standards before they can be integrated into the RCI [70,73].

## 8. Conclusions

The cardiometabolic consequences of diet and physical activity cannot be fully explained by their average levels [2]. Individuals with similar daily energy intake and comparable MVPA may differ substantially in vascular stiffness and glycemic variability because of differences in their temporal behavioral patterns. When eating, physical activity, and sleep rhythms are aligned across the 24 h cycle, peripheral clocks may be more effectively entrained. This alignment may help preserve the diurnal expression of eNOS, improve the correspondence between β-cell insulin secretory peaks and energy intake, and maintain nocturnal blood pressure dipping [14,15]. Conversely, fragmented or misaligned rhythms may disrupt these protective processes. The proposed Rhythm Coherence Index (RCI) is a hypothesis-generating candidate composite for studying temporal alignment across these three behavioral rhythm layers; it is not an existing validated exposure metric.

This review makes three principal contributions. First, it synthesizes eating, physical activity, and sleep rhythms within a single operational construct—rhythm coherence—and links this construct to two outcome domains. Vascular endpoints include PWV, FMD, and ABPM-derived measures. Glycemic endpoints include TIR, coefficient of variation, and mean amplitude of glycemic excursions. In contrast to prior reviews of chrononutrition, time-restricted eating, and exercise snacks, which have generally focused on a single behavioral rhythm or outcome domain, the rhythm coherence framework integrates both behavioral and outcome dimensions. Second, the proposed RCI identifies within-rhythm regularity and cross-rhythm coupling as candidate domains, thereby providing a methodological hypothesis for future observational and intervention studies rather than a finalized score. Third, the athlete-focused analysis identifies a rhythm coherence paradox: despite superior fitness, high training volumes may disrupt eating, physical activity, and sleep rhythms. Flexible multimodal biosensors may provide the phenotyping tools required to investigate this paradox in precision sport settings.

As wearable technologies progress from CGM and actigraphy to flexible, multi-analyte biosensors capable of assessing glucose, electrolytes, cortisol, and muscle activation, continuous observation of 24 h behavioral and biochemical patterns under free-living conditions may become feasible. Only after staged prospective validation could an RCI be considered for real-time estimation or evaluation within just-in-time adaptive interventions. At present, rhythm coherence should be viewed as a proposed research framework, not as a risk factor or decision signal ready to be communicated to patients, clinicians, or coaches. The integration of precision nutrition, precision exercise, and precision sport within the rhythm coherence framework is increasingly technically plausible. Its clinical relevance, however, remains to be established through prospective validation and intervention studies.

## Figures and Tables

**Table 1:** Coreconstructs and operational metrics of rhythm coherence.

Construct	Conceptual Definition	Core Operational Metrics	Data Source	Direction in RCI
Circadian misalignment	Discordance between endogenous circadian phase and behavioral timing [14,15,16].	DLMO, core body-temperature nadir, actigraphy-derived phase angle.	Melatonin, temperature, actigraphy	Inverse construct; larger phase mismatch indicates lower coherence.
Chrononutrition disruption	Instability or biological mistiming of the eating rhythm [5,8,9,10,11,13,17,18,19,20].	Eating-window start/end, night-eating frequency, meal timing variability.	Meal log, EMA, CGM-assisted inference	Negative component when eating is late, unstable, or extended.
Chronotype mismatch/social jetlag	Mismatch between individual circadian preference and imposed social schedule [12,21].	MCTQ/MEQ; difference between midsleep on workdays and free days.	Questionnaire, sleep diary, actigraphy	Trait/schedule modifier; not equivalent to day-level coherence.
Rest–activity rhythm fragmentation	Instability or fragmentation of the 24 h rest–activity rhythm/pattern [6,22,23,24].	IS, IV, RA, MVPA acrophase.	Actigraphy or wearable accelerometry	Low IS/RA and high IV indicate lower coherence.
Meal–activity coupling	Temporal alignment between meals and postprandial physical activity [7,19,25,26,27].	Light activity or MVPA within 30–60 min after meals; meal-to-activity lag.	Meal log + actigraphy	Positive cross-rhythm component.
Rhythm coherence/RCI	Temporal stability and alignment across eating, activity, and rest–sleep rhythms [7]; proposed construct.	Standardized composite of meal jitter, eating-window regularity, IS/IV/RA, SRI, meal–activity coupling, and phase alignment.	Multimodal phenotyping	Hypothetical direction only: greater coherence would be expected to yield a higher candidate value; no validated score, range, or threshold exists.

Note: The constructs, operational metrics, and directions shown in Table 1 are hypothesis-generating examples for future testing; they are not validated RCI components, weights, scoring rules, thresholds, or clinical interpretations. RCI = Rhythm Coherence Index; DLMO = dim-light melatonin onset; EMA = ecological momentary assessment; CGM = continuous glucose monitoring; MCTQ = Munich Chronotype Questionnaire; MEQ = Morningness–Eveningness Questionnaire; IS = interdaily stability; IV = intradaily variability; RA = relative amplitude; MVPA = moderate-to-vigorous physical activity; SRI = Sleep Regularity Index.

**Table 2:** Measurement framework for multimodal rhythm-coherence phenotyping.

Signal Layer	Modality	Core Metrics for RCI or Validation	Temporal Scale	Primary Role	Key Measurement Caveat
Eating rhythm	Digital meal logging, EMA, photo-assisted records	Eating-window start/end, meal timing variability, eating frequency, macronutrient timing.	Event-level; ideally ≥7 days.	Exposure layer.	Participant burden and underreporting; image-based estimates vary by food type [48,49,50,51,52].
Activity rhythm	Research-grade accelerometry or wearable actigraphy	MVPA, sedentary bouts, IS, IV, RA, activity acrophase.	30–60 s epochs; usually 7–14 days.	Exposure layer and coupling metric.	Placement and algorithm choice affect sedentary time and MVPA classification [6,22,23,24,53,54].
Rest–sleep rhythm	Actigraphy, sleep diary, validated sleep algorithms	Sleep onset/offset, midsleep variability, SRI, social jetlag.	Nightly; ideally ≥7 days.	Rest–sleep layer.	Actigraphy estimates sleep timing better than sleep architecture [12,21,24,33,34,50,53,55,56].
Metabolic readout	CGM	TIR, CV%, MAGE, postprandial glucose AUC.	1–15 min sampling; 7–14 days.	Outcome and meal-response validation layer.	Sensor bias may be larger in euglycemic ranges; dietary composition is not captured [25,50,57,58,59,60,61,62,63,64,65,66].
Vascular readout	ABPM, PWV, FMD	24 h BP, nocturnal dipping, morning surge, arterial stiffness, endothelial function.	ABPM 15–30 min; PWV/FMD visit-based.	Clinical outcome layer.	ABPM may disturb sleep; PWV/FMD are less suited to dense free-living sampling [6,8,17,41,44,45,46,47,67,68,69,70,71,72].
Biochemical/autonomic layer	Flexible sweat, EMG, skin-temperature, and NIRS sensors	Electrolytes, lactate, cortisol, muscle activation, thermal/autonomic proxies.	Continuous or repeated high-resolution sampling.	Mechanistic and athlete-focused layer.	Mostly research-grade; calibration and regulatory validation remain incomplete [7,54,70,71,72,73].

Note: ABPM = ambulatory blood pressure monitoring; AUC = area under the curve; BP = blood pressure; CV = coefficient of variation; EMG = electromyography; FMD = flow-mediated dilation; MAGE = mean amplitude of glycemic excursions; NIRS = near-infrared spectroscopy; PWV = pulse wave velocity; TIR = time in range.

**Table 3:** Evidence domains linking 24 h behavioral rhythms with vascular and glycemic outcomes.

Exposure Domain	Evidence Base	Primary Outcomes	Overall Pattern	Interpretation for RCI Framework
Eating window and time-restricted eating	RCTs and recent systematic reviews/meta-analyses	BP, TIR, glycemic variability, body weight	Earlier or shorter eating windows generally show more favorable cardiometabolic profiles than late or prolonged windows.	Supports eating timing as a core within-rhythm component [11,13,17,18,20,31,32,38,68,75,76,77,78,79,80,81].
Meal timing variability	CGM + meal-log observational studies	CV%, MAGE, postprandial glucose AUC	Greater meal timing variability is associated with greater glycemic variability, often beyond eating-window length.	Supports meal jitter as a negative RCI component [5,7,8,9,17,19,35,36,40,61,63,65,81].
Postprandial activity and sedentary breaks	Randomized crossover trials and meta-analyses	Postprandial glucose AUC, TIR	Brief walking or activity breaks after meals reduce postprandial glucose excursions.	Supports meal–activity coupling as a cross-rhythm component [25,26,27,58,66].
Exercise timing	RCTs and prospective cohorts	BP, arterial stiffness, glycemic control	Morning and evening exercise may produce different vascular and glycemic effects; effects may depend on chronotype and metabolic status.	Supports phase-sensitive weighting rather than total MVPA alone [28,29,37,59,60].
Sleep regularity and rest–activity fragmentation	Actigraphy-based observational cohorts	PWV, BP rhythm, glycemic variability	Irregular sleep and fragmented rest–activity rhythms are linked to adverse vascular and metabolic profiles.	Supports rest–sleep stability as an independent RCI layer [12,21,24,33,34,39,43,46,47,53,55,56].
Multimodal rhythm coherence	Multimodal phenotyping and proposed composite models	PWV, TIR, CV%, MAGE	Joint eating–activity–sleep metrics appear to explain risk beyond single-modality measures.	Hypothesis-generating; requires prospective validation of RCI against clinical outcomes [7]; hypothesis-generating.

## Data Availability

No new data were created or analyzed in this study. Data sharing is not applicable to this article.

## Supplementary Materials

The following supporting information can be downloaded at https://www.mdpi.com/article/10.3390/nu18152546/s1: File S1: Transparent evidence identification and selection methods; Table S1: Database record ledger: original retained PubMed/Web of Science exports and Scopus reproducibility-rerun exports; Table S2: Scopus Reproducibility Search Run (20 July 2026); Figure S1: Evidence-identification, screening, synthesis, and citation-selection workflow. PubMed, Scopus, and Web of Science are shown as information sources. The successive stages distinguish 303 candidate records, 131 high-priority records used in full-text narrative synthesis, and 92 sources directly cited in the final manuscript.

## Abbreviations

**Abbreviation**	**Definition**
ABPM	Ambulatory blood pressure monitoring
CGM	Continuous glucose monitoring
CV%	Coefficient of variation
eNOS	Endothelial nitric oxide synthase
FMD	Flow-mediated dilation
HRV	Heart rate variability
IS/IV/RA	Interdaily stability/intradaily variability/relative amplitude
JITAI	Just-in-time adaptive intervention
MAGE	Mean amplitude of glycemic excursions
MVPA	Moderate-to-vigorous physical activity
PWV	Pulse wave velocity
RCI	Rhythm Coherence Index
RED-S	Relative Energy Deficiency in Sport
SCN	Suprachiasmatic nucleus
SRI	Sleep Regularity Index
TIR	Time in range
TRE	Time-restricted eating
