# Supplementary material for: Dietary Behavior, Physical Activity, and 24-Hour Rhythm Coherence: A Digital Phenotyping Perspective on Vascular and Glycemic Health
Source: Nutrients. 2026 Aug 4;18(15):2546. doi: 10.3390/nu18152546 (PMC13468063; doi:10.3390/nu18152546)
Supplement: Supplementary file 1 [file nutrients-18-02546-s001.zip › nutrients-4401541-supplementary.pdf]

Supplementary File S1

Transparent Evidence Identification and Selection Methods

Purpose and Review Design

This supplement documents the transparent evidence-identification and selection procedures used to support a semi-structured narrative review titled "Dietary Behavior, Physical Activity, and 24-Hour Rhythm Coherence: A Digital Phenotyping Perspective on Vascular and Glycemic Health." The purpose of this process was to identify and organize relevant evidence for narrative synthesis; it was not designed as a systematic review or meta-analysis. Accordingly, the process was not preregistered, duplicate independent screening and inter-rater reliability assessment were not undertaken, and no formal risk-of-bias assessment was performed.

Information Sources and Search Dates

The evidence-identification process used PubMed, Scopus, and Web of Science Core Collection. The retained PubMed and Web of Science export files were generated on 14 May 2026. The database date filters were set from 1 January 2020 to 31 May 2026; the effective coverage ended on the search date. Scopus results were searched using equivalent TITLE-ABS-KEY syntax and were merged into the master citation library during the original workflow, before a separate source-level export count was preserved. Foundational studies published before 2020 could be added purposively for conceptual or mechanistic context and were not counted in the staged screening flow. Targeted athlete-focused searches used a broader 2018–2026 window.

The retained source-tagged exports from the original evidence-identification workflow comprised 1,508 records (771 from PubMed and 737 from Web of Science). Scopus was also searched, but the original pre-merge Scopus source count and record-level database attribution were not retained. To document reproducibility without retrospectively altering the original screening cohort, the Scopus strategy was rerun on 20 July 2026. The rerun counts are reported in Tables S1–S2 and were not added to the original screening flow.

Deduplication

Records from PubMed, Scopus, and Web of Science were resolved using normalized DOI exact matches; PMID, Scopus EID, or Web of Science accession matches when DOI was unavailable; normalized title, year, and first-author matches; and a secondary normalized title-plus-year merge when identifiers were incomplete. For duplicate groups, the richest record was retained and missing fields were merged where available. The original cross-database workflow produced 1,081 unique records. Because the original pre-merge Scopus source attribution was not retained, its incremental contribution to that historical cohort cannot be reconstructed. The 20 July 2026 Scopus rerun is therefore reported separately as a reproducibility audit rather than treated as a retrospective addition to the original cohort.

Staged Screening and Eligibility Criteria

Pass 1: Broad Relevance Screen

At the broad screen, a record was retained if it concerned an adult human study, review, consensus statement, or methodological paper with at least one relevant behavioral exposure (eating timing/window/frequency/jitter; activity timing/fragmentation; sleep regularity/circadian misalignment; or free-living digital phenotyping of these behaviors) and a vascular, glycemic, cardiometabolic, or relevant measurement outcome. Broad-screen exclusions were animal-only studies, pediatric-only studies, pharmacological chronotherapy as the main focus, non-English reports without a translated abstract, very limited conference abstracts, pure device-engineering reports without human application, and studies clearly outside the review scope.

Of 1081 unique records, 619 were directly retained. A further 322 records had no usable abstract and underwent conservative title-based adjudication. A record was retained at this step only when the title contained both a relevant exposure signal and a relevant outcome signal; 75 records were retained and 247 were not carried forward. The remaining broad-screen exclusions comprised 134 clearly out-of-scope records and 6 animal-only records set aside from the human evidence pool. This resulted in 694 records for strict screening.

Pass 2: Strict Relevance Screen

The strict screen required both (i) a relevant behavioral rhythm/timing/digital-phenotyping exposure and (ii) a primary vascular or glycemic outcome.

Principal reason for not carrying forward	n
No rhythm or timing exposure	149
No vascular or glycemic primary outcome	65
Pediatric-only population	41
Mental-health focus without strict endpoint	40
Weight/body-composition outcomes only	36
Insufficiently informative abstract/title	29
Protocol-only report	27
Lipid/inflammation-only outcome	20
Device validation without outcome data	18
Animal-only study	12
Total not carried forward	437

Of 694 strict-screen records, 223 were retained and 34 were retained as limited-information but potentially relevant records, yielding a core candidate pool of 257 records.

Targeted Section 6 Supplemental Search

To strengthen the athlete/highly active subsection, targeted PubMed, Scopus, and Web of Science searches used three athlete-focused strings: S6a (athlete rhythm/meal timing and cardiometabolic outcomes), S6b (athlete vascular function), and S6c (RED-S/energy availability and cardiovascular outcomes). The retained source-tagged PubMed and Web of Science exports comprised 168 records. Scopus was included in the original merged workflow, but its original record-level source attribution was not retained; its incremental contribution to the

retained athlete set therefore cannot be reconstructed. After duplicate resolution and targeted assessment, 46 records (44 include; 2 borderline) were retained and merged with the core candidate pool.

Candidate Evidence Pool and Prioritization

The final candidate evidence pool comprised 303 records: 257 from the core strict screen plus 46 from the targeted Section 6 supplemental search. These records supported topic mapping and prioritization. Following full-text relevance assessment, 131 high-priority records were used in the narrative synthesis. Of these and the relevant foundational/contextual sources, 92 sources were cited directly in the final manuscript.

Priority	Operational definition	n
High	Direct evidence linking a behavioral rhythm, timing, or digital-phenotyping exposure to a vascular or glycemic endpoint; includes athlete vascular-risk evidence used in Section 6.	182
Medium	Strong relevance to one framework component with partial/indirect support for the other component; or an interpretive review/methods source.	119
Low	Contextual background value only.	2

Priority labels represent relevance and evidentiary utility, not a formal study-quality rating, certainty-of-evidence judgment, or risk-of-bias assessment.

The 303-record candidate pool was used for topic mapping and prioritization. Full-text relevance assessment retained 131 high-priority records for narrative synthesis. Citation selection was then guided by direct section-specific relevance, study design, population, endpoint, and recency; 92 sources were cited directly in the manuscript. Thus, 303 denotes the candidate pool, 131 denotes records synthesized at full-text level, and 92 denotes sources directly cited.

Limitations of the Evidence-Identification Process

- The strategy was intentionally structured but not systematic-review compliant.
- It used three databases. The original pre-merge Scopus source attribution was not retained; therefore, the 20 July 2026 Scopus rerun is reported separately and is not used to recalculate the historical screening cohort.
- It did not include protocol registration, duplicate independent screening, a formal risk-of-bias instrument, GRADE assessment, or quantitative evidence synthesis.
- These limitations are acknowledged to avoid overinterpreting the workflow as a formal systematic review.

Full Concept Strings

Main-search concept strings (2020-01-01 to 2026-05-31)

S1: ("circadian misalignment" OR "chrono-nutrition" OR "chrono-exercise" OR "meal timing" OR "time-restricted eating" OR "rhythm coherence" OR "behavioral rhythm") AND ("arterial stiffness" OR "endothelial function" OR "blood pressure" OR "glycemic variability" OR cardiometabolic)

S2: ("digital phenotyping" OR "multimodal sensing" OR "continuous glucose monitoring" OR wearable OR actigraphy) AND ("dietary behavior" OR "physical activity" OR "meal timing") AND (cardiometabolic OR vascular OR glycemic)

S3: ("24-hour composition" OR "compositional analysis" OR "behavioral phenotype" OR "eating jitter" OR "activity fragmentation") AND (cardiovascular OR metabolic)

S4: (athlete OR "elite sport" OR "endurance training" OR "master athlete") AND (circadian OR "meal timing" OR "sleep regularity") AND ("arterial stiffness" OR "blood pressure" OR "RED-S" OR vascular)

S4a: (athlete OR "elite sport" OR "endurance training" OR "master athlete" OR "competitive athlete") AND (circadian OR "meal timing" OR "sleep regularity" OR chrono OR "time-restricted")

S4b: (athlete OR "master athlete" OR "endurance athlete") AND ("arterial stiffness" OR "blood pressure" OR vascular OR "RED-S" OR cardiometabolic)

S5: ("flexible biosensor" OR "wearable biosensor" OR "sweat sensor" OR "epidermal sensor" OR "cuffless blood pressure") AND (nutrition OR "physical activity" OR cardiometabolic)

PubMed implementation: terms were searched in Title/Abstract fields with the publication-date limiter. Web of Science Core Collection implementation: TS=(concept string) AND PY=(2020-2026). Scopus implementation for S1–S5 used TITLE-ABS-KEY field searching with PUBYEAR > 2019 AND PUBYEAR < 2027. The same Boolean concepts used in PubMed and Web of Science were retained; the complete database-specific expressions are listed in Supplementary File S1. For any run with more than 200 records, the first 200 records ranked by database relevance were exported.

Targeted Section 6 supplemental searches (2018-01-01 to 2026-05-31)

S6a PubMed: (("athlete"[Title/Abstract] OR "endurance athlete"[Title/Abstract] OR "master athlete"[Title/Abstract] OR "elite athlete"[Title/Abstract] OR "competitive sport"[Title/Abstract] OR "trained athlete"[Title/Abstract]) AND ("time-restricted eating"[Title/Abstract] OR "meal timing"[Title/Abstract] OR "intermittent fasting"[Title/Abstract] OR "chrono"[Title/Abstract] OR "eating window"[Title/Abstract] OR "circadian"[Title/Abstract]) AND ("blood pressure"[Title/Abstract] OR "arterial stiffness"[Title/Abstract] OR "vascular"[Title/Abstract] OR "glucose"[Title/Abstract] OR "glycemic"[Title/Abstract] OR "cardiometabolic"[Title/Abstract] OR "cardiovascular"[Title/Abstract])) AND ("2018/01/01"[PDat]:"2026/05/31"[PDat])

S6a Web of Science: TS=(("athlete" OR "endurance athlete" OR "master athlete" OR "elite athlete" OR "competitive sport" OR "trained athlete") AND ("time-restricted eating" OR "meal timing" OR "intermittent fasting" OR "chrono" OR "eating window" OR "circadian") AND ("blood pressure" OR "arterial stiffness" OR "vascular" OR "glucose" OR "glycemic" OR "cardiometabolic" OR "cardiovascular")) AND PY=(2018-2026)

S6b PubMed: (("athlete"[Title/Abstract] OR "endurance athlete"[Title/Abstract] OR "master athlete"[Title/Abstract] OR "elite athlete"[Title/Abstract] OR "highly trained"[Title/Abstract]) AND ("arterial stiffness"[Title/Abstract] OR "pulse wave velocity"[Title/Abstract] OR "endothelial function"[Title/Abstract] OR "flow-mediated dilation"[Title/Abstract] OR "FMD"[Title/Abstract] OR "PWV"[Title/Abstract] OR "arterial compliance"[Title/Abstract])) AND ("2018/01/01"[PDat]:"2026/05/31"[PDat])

S6b Web of Science: TS=(("athlete" OR "endurance athlete" OR "master athlete" OR "elite athlete" OR "highly trained") AND ("arterial stiffness" OR "pulse wave velocity" OR "endothelial function" OR "flow-mediated dilation" OR "FMD" OR "PWV" OR "arterial compliance")) AND PY=(2018-2026)

S6c PubMed: (("athlete"[Title/Abstract] OR "sport"[Title/Abstract]) AND ("RED-S"[Title/Abstract] OR "relative energy deficiency"[Title/Abstract] OR "energy availability"[Title/Abstract] OR "athlete triad"[Title/Abstract]) AND ("cardiovascular"[Title/Abstract] OR "vascular"[Title/Abstract] OR "blood pressure"[Title/Abstract] OR "cardiometabolic"[Title/Abstract])) AND ("2018/01/01"[PDat]:"2026/05/31"[PDat])

S6c Web of Science: TS=(("athlete" OR "sport") AND ("RED-S" OR "relative energy deficiency" OR "energy availability" OR "athlete triad") AND ("cardiovascular" OR "vascular" OR "blood pressure" OR "cardiometabolic")) AND PY=(2018-2026)

The S6a–S6c searches were implemented in PubMed, Scopus, and Web of Science Core Collection. The retained source-tagged PubMed and Web of Science exports comprised 168 records before duplicate resolution (PubMed: 2, 30, and 32; Web of Science: 4, 51, and 49, respectively). Scopus implementation for S6a–S6c used TITLE-ABS-KEY field searching with PUBYEAR > 2017 AND PUBYEAR < 2027; the complete athlete-focused expressions are listed in Supplementary File S1. The original Scopus source-level counts and record-level attribution were not retained after merging. In the 20 July 2026 rerun, S6a, S6b, and S6c returned 80, 207, and 81 records, of which 80, 200, and 81 were exported, respectively. These rerun records were documented for reproducibility and were not retrospectively added to the original athlete-focused screening set.

Scopus database-specific search strings

S1: TITLE-ABS-KEY(("circadian misalignment" OR "chrono-nutrition" OR "chrono-exercise" OR "meal timing" OR "time-restricted eating" OR "rhythm coherence" OR "behavioral rhythm") AND ("arterial stiffness" OR "endothelial function" OR "blood pressure" OR "glycemic variability" OR cardiometabolic)) AND PUBYEAR > 2019 AND PUBYEAR < 2027

S2: TITLE-ABS-KEY(("digital phenotyping" OR "multimodal sensing" OR "continuous glucose monitoring" OR

wearable OR actigraphy) AND ("dietary behavior" OR "physical activity" OR "meal timing") AND (cardiometabolic OR vascular OR glycemic)) AND PUBYEAR > 2019 AND PUBYEAR < 2027

S3: TITLE-ABS-KEY(("24-hour composition" OR "compositional analysis" OR "behavioral phenotype" OR "eating jitter" OR "activity fragmentation") AND (cardiovascular OR metabolic)) AND PUBYEAR > 2019 AND PUBYEAR < 2027

S4: TITLE-ABS-KEY((athlete OR "elite sport" OR "endurance training" OR "master athlete") AND (circadian OR "meal timing" OR "sleep regularity") AND ("arterial stiffness" OR "blood pressure" OR "RED-S" OR vascular)) AND PUBYEAR > 2019 AND PUBYEAR < 2027

S4a: TITLE-ABS-KEY((athlete OR "elite sport" OR "endurance training" OR "master athlete" OR "competitive athlete") AND (circadian OR "meal timing" OR "sleep regularity" OR chrono OR "time-restricted")) AND PUBYEAR > 2019 AND PUBYEAR < 2027

S4b: TITLE-ABS-KEY((athlete OR "master athlete" OR "endurance athlete") AND ("arterial stiffness" OR "blood pressure" OR vascular OR "RED-S" OR cardiometabolic)) AND PUBYEAR > 2019 AND PUBYEAR < 2027

S5: TITLE-ABS-KEY(("flexible biosensor" OR "wearable biosensor" OR "sweat sensor" OR "epidermal sensor" OR "cuffless blood pressure") AND (nutrition OR "physical activity" OR cardiometabolic)) AND PUBYEAR > 2019 AND PUBYEAR < 2027

S6a: TITLE-ABS-KEY((athlete OR "endurance athlete" OR "master athlete" OR "elite athlete" OR "competitive sport" OR "trained athlete") AND ("time-restricted eating" OR "meal timing" OR "intermittent fasting" OR chrono OR "eating window" OR circadian) AND ("blood pressure" OR "arterial stiffness" OR vascular OR glucose OR glycemic OR cardiometabolic OR cardiovascular)) AND PUBYEAR > 2017 AND PUBYEAR < 2027

S6b: TITLE-ABS-KEY((athlete OR "endurance athlete" OR "master athlete" OR "elite athlete" OR "highly trained") AND ("arterial stiffness" OR "pulse wave velocity" OR "endothelial function" OR "flow-mediated dilation" OR FMD OR PWV OR "arterial compliance")) AND PUBYEAR > 2017 AND PUBYEAR < 2027

S6c: TITLE-ABS-KEY((athlete OR sport) AND ("RED-S" OR "relative energy deficiency" OR "energy availability" OR "athlete triad") AND (cardiovascular OR vascular OR "blood pressure" OR cardiometabolic)) AND PUBYEAR > 2017 AND PUBYEAR < 2027

Audit Trail Retained by the Authors

Search strings, retained PubMed and Web of Science run logs, deduplication reports, broad-screen decisions, strict-screen decisions, and Section 6 supplemental-screen decisions are retained by the authors and can be made available upon reasonable request. The absence of a separate pre-merge Scopus export count is disclosed as a source-record limitation.

Table S1. Database record ledger: original retained PubMed/Web of Science exports and Scopus reproducibility-rerun exports.

String	Concept	PubMed	Web of Science	Scopus rerun†
S1	Rhythm/timing/circadian exposures AND vascular or glycemic outcomes	200	200	200
S2	Digital phenotyping/wearables AND diet, activity, or meal timing AND cardiometabolic outcomes	200	150	200
S3	24-hour composition or behavioral phenotypes AND cardiovascular or metabolic outcomes	91	109	200
S4	Athlete AND circadian/meal timing/sleep regularity AND vascular/RED-S outcomes	0	4	24
S4a	Athlete AND circadian, meal timing, sleep regularity, chrono, or time restriction	65	50	200
S4b	Athlete AND arterial stiffness, blood pressure, vascular, RED-S, or cardiometabolic outcomes	200	200	200
S5	Flexible or wearable biosensors AND nutrition, physical activity, or cardiometabolic outcomes	15	24	98
Total	Column totals; Scopus total is reproducibility-rerun only	771	737	1,122

Note: S4a and S4b were relaxed athlete-focused searches used after the original three-layer athlete string (S4) was too restrictive. The Scopus column reports records exported during the reproducibility rerun conducted on 20 July 2026; these records were not retrospectively added to the original screening cohort. The original pre-merge Scopus contribution cannot be reconstructed because source-level attribution was not retained. The Web of Science S4a/S4b timestamps were not entered contemporaneously, although the exported files were included in deduplication.

Table S2. Scopus Reproducibility Search Run (20 July 2026)

Search	Year window	Total results	Records exported
S1	2020–2026	506	200
S2	2020–2026	752	200
S3	2020–2026	302	200
S4	2020–2026	24	24
S4a	2020–2026	487	200
S4b	2020–2026	2013	200
S5	2020–2026	98	98
S6a	2018–2026	80	80
S6b	2018–2026	207	200
S6c	2018–2026	81	81

Note: The Scopus rerun verified that the database-specific syntax was executable. Searches overlap substantially; therefore, run totals and exports are not summed. The rerun is reported as a reproducibility audit and was not added as a new cohort to the original staged screening workflow. In the original workflow, Scopus records were merged during cross-database deduplication; because record-level source attribution was not retained, the database's incremental contribution cannot be reconstructed.

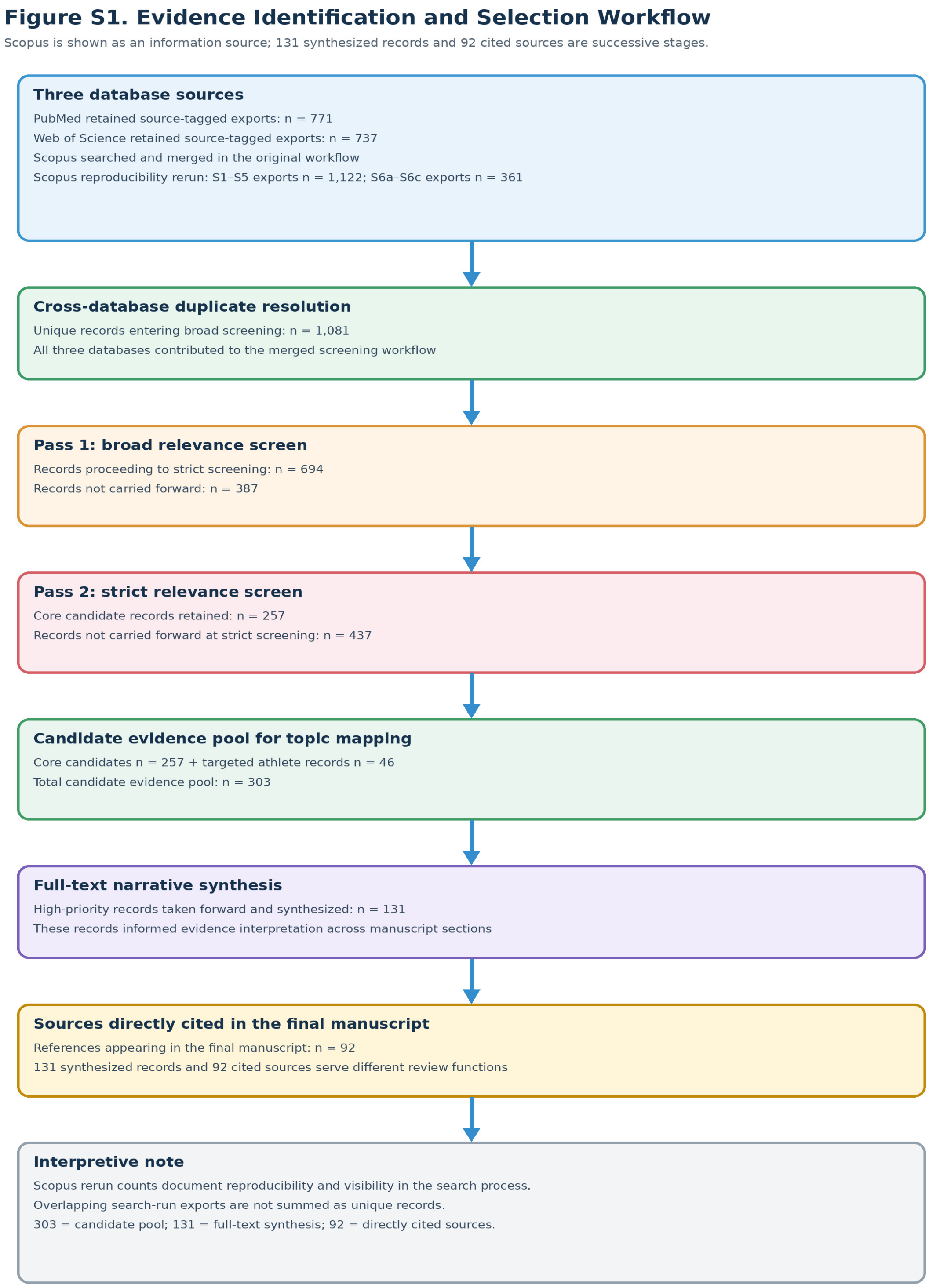

Figure S1. Evidence-identification, screening, synthesis, and citation-selection workflow. PubMed, Scopus, and Web of Science are shown as information sources. The successive stages distinguish 303 candidate records, 131 high-priority records used in full-text narrative synthesis, and 92 sources directly cited in the final manuscript.
